# Long-term spatiotemporal changes in nitrate contamination of municipal groundwater resources after sewerage network construction in the Hungarian Great Plain

**DOI:** 10.1007/s11356-024-35280-9

**Published:** 2024-10-15

**Authors:** Tamás Mester, György Szabó, Emőke Kiss, Dániel Balla

**Affiliations:** 1 Department of Landscape Protection and Environmental Geography, Faculty of Science and Technology, University of Debrecen, Debrecen, H‑4032 Hungary; 2 Department of Data Science and Visualization, Faculty of Informatics, University of Debrecen, Debrecen, H‑4028 Hungary

**Keywords:** Nitrate, Nitrate Pollution Index, Groundwater quality, Pollution, Wastewater, Sewerage network

## Abstract

Over the last decades, as a consequence of wastewater discharges and other anthropogenic sources, severe nitrate (NO_3_^−^) pollution has developed in municipal environment causing global concern. Thus, eliminating the potential sources of pollution is one of the major challenges of the twenty-first century, whereby sanitation services are essential for ensuring public health and environmental protection. In the present study, long-term monitoring (2011–2022) of shallow groundwater NO_3_^−^ contamination in municipal environment was carried following the construction of the sewerage network (2014) in the light of the pre-sewerage situation. Our primary aim was to assess the long-term effects of sewerage on nitrate NO_3_^−^ levels in the shallow groundwater and evaluate the efficiency of these sanitation measures over time. Based on the results, significant pollution of the shallow groundwater in the municipality was identified. During the pre-sewer period, NO_3_^−^ concentrations exceeded the 50 mg/L limit in the majority of monitoring wells significantly, upper quartile values ranged between 341 and 623 mg/L respectively. Using Nitrate Pollution Index (NPI) and interpolated NO_3_^−^ pollution maps, marked spatial north–south differences were detected. In order to verify the presence of wastewater discharges in the monitoring wells, the isotopic ratio shifts (δ) for ^18^O and D(^2^H) were determined, confirming municipal wastewater effluent. Variations in NO_3_^−^/Cl^−^ molar ratios suggest also contamination from anthropogenic sources, including septic tank effluent from households and the extensive use of manure. Data series of 7 years (2015–2022) after the investment indicate marked positive changes by the appearance of decreasing trends in NO_3_^−^ values confirmed by Wilcoxon signed rank test and ANOVA. By comparing the pre- and post-sewerage conditions, the mean NO_3_^−^ value decreased from 289.7 to 175.6 mg/L, with an increasing number of monitoring wells with concentrations below the limit. Our results emphasise the critical role of sanitation investments, while also indicating that the decontamination processes occur at a notably slow pace. Detailed, long-term monitoring is therefore essential to ensure accurate follow-up of the ongoing changes. The results can provide information for local citizens and authorities to improve groundwater management tools in the region.

## Introduction

Human activities in built-up areas generate significant pollution loads, resulting in adverse consequences on groundwater quality especially in shallow aquifers (Fathmawati et al. 2018; Huerfano-Moreno et al. 2023). Over the last decades, urbanisation and linked human activities altered the groundwater quality and quantity worldwide (He et al. 2022; Huang et al. 2020; Zhang et al. 2019). In rural areas, various sources endanger the municipal groundwater resources, the most crucial of these are uninsulated septic tanks, pit latrines, inadequate sewage treatment, landfills without proper engineering controls, animal husbandry with manure storage and other non-point sources trough leaching like garden fertilisation and agricultural activities around the settlements (Ahmad and Ghanem 2021; Roba et al. 2021). Eliminating these potential sources of pollution is one of the major challenges of the twenty-first century, even in more developed countries.

Urbanisation, particularly through the development of sewerage systems, has been identified as a significant factor influencing groundwater quality (Hashmi et al. 2023; Huang et al. 2020). Studies have shown that uninsulated septic systems and untreated wastewater are major contributors to elevated nitrate levels in shallow aquifers, particularly in peri-urban and rural areas (He et al. 2022). Results indicate that while the construction of sewerage networks mitigates nitrate pollution by reducing wastewater infiltration into the subsurface, the clean-up processes are gradual, with rates of improvement varying due to factors such as soil characteristics and local hydrogeology (Zhang et al. 2020).

Despite short-term reductions in nitrate concentrations following sewerage interventions, the long-term effects require sustained monitoring to understand the persistence and extent of improvements in groundwater quality. Studies demonstrate that while sewerage systems contribute to a decline in nitrate concentrations, the reductions are often slow and influenced by local hydrological conditions and urban infrastructure (Gan et al. 2022; Liu et al. 2024). Nitrate (NO_3_^−^) is identified as one of the most common contaminants in groundwater, specifically in shallow aquifers worldwide by its high solubility and low fixation in soils (Arumi et al. 2005; Bhatnagar and Sillanpää, 2011). Nitrogen (N) is present in small amounts in many environments, with the vast majority originating from the atmosphere and soil, which can be significantly elevated by anthropogenic sources. When N is released into the soil, it undergoes the main processes of fixation, assimilation, ammonification, nitrification and denitrification (Burger and Jackson 2003). Leaching of NO_3_^−^ from the unsaturated zone is a complex interaction of several factors, such as land use practices, soil nitrogen loading, groundwater recharge and water table depth (Birkinshaw and Ewen 2000). The spatial–temporal occurrence of nitrate in groundwater depends according to Almasri (2007) on on-ground nitrogen loading, soil characteristics and groundwater properties.

Although high NO_3_^−^ loads occur mainly due to diffuse sources of pollution worldwide, point sources can result in extremely high NO_3_^−^ concentrations in localised areas (Zhou et al. 2015). In addition, there are significant regional differences in terms of the main sources. While in the Mediterranean region, nitrate pollution is mainly caused by the use of nitrogen-rich fertilisers, in developing countries, the main source of groundwater nitrate pollution is the lack of proper conditions of sanitation (Kapembo et al. 2016; Re et al. 2017).

Since NO_3_^−^ pollution is the most common form of water pollution globally, it is of strategic importance to monitor its spatial and temporal evolution (Abascal et al. 2022). Numerous studies have been carried out over the last few years to investigate NO_3_^−^ pollution of groundwater resources. A number of methods have been used by environmental scientists around the world to evaluate water contamination, some of which include Water Quality Index methods, Nemerov Index, multivariate statistical analysis, fuzzy mathematics and GIS techniques (Jha et al. 2020; Mester et al. 2021; Petrushka et al. 2023; Yang et al. 2023). The Water Quality Index (WQI) method is widely used to determine the overall quality and suitability for drinking (Franz et al. 2022; Han et al. 2023; Mester et al. 2020a, b). The Nitrate Pollution Index (NPI) is special, single-parameter Water Quality Index, which can be applied to indicate the anthropogenic origin of nitrate in groundwater (Bahrami et al. 2020). Several studies have used NPI to assess whether nitrate originates from human activities (El Mountassir et al. 2022; Kada et al. 2023). Based on the high NPI values in Sidi Slimane region, Morocco, the authors suggested regular water quality monitoring in the future (Al-Aizari et al. 2023). Over recent years, groundwater quality has been assessed and monitored on a regular basis using a Geographic Information System (GIS), complemented by the IDW interpolation method, which has proven to be an effective tool for assessing and analysing spatial information on water resources (Balla et al. 2023; Ram et al. 2021). In the present study, GIS technique IDW interpolation method has been used for spatiotemporal assessment of NO_3_^−^ contamination of the shallow groundwater.

Long-term monitoring data are of paramount importance to assess environmental changes, during which numerous studies have used interpolation techniques to evaluate long-term monitoring data and provide more detailed analyses. Chaudhuri et al. (2012) and colleagues assessed the spatio-temporal changes of groundwater NO_3_^−^ levels in TX, USA, between 1960 and 2010, which revealed a deterioration in groundwater quality, as well as the other important finding that the main limitation to assessing groundwater NO_3_^−^ concentrations was the lack of recent and adequate monitoring data. Results of the study conducted by Kim and colleagues (2018) on the Korean island of Jeju from 1993 to 2015 showed that declining nitrate levels in protected areas are a successful result of environmental protection measures (Kim et al. 2003). High NO_3_^−^ concentrations in drinking water sources can pose a potential environmental and public health risk. Nitrates can affect human health by causing methemoglobinaemia “blue-baby syndrome” especially in infants and thyroid effects (Shaban et al. 2023; Ward et al. 2005). In addition, several studies demonstrated the carcinogenic feature of N-nitroso compounds and the relation with gastric cancer (Picetti et al. 2022; Sandor et al. 2001). It is important to note that although groundwater nitrate concentrations are within the World Health Organization (WHO) drinking water standard of 50 mg/L, they still pose a non-carcinogenic health risk to humans, especially children and infants (Qasemi et al. 2022).

These adverse effects have contributed to the numerous human health risk assessment studies that have been carried out in recent years (Adimalla and Qian 2023; Liu et al. 2022; Sheng et al. 2023). Alsabti and colleagues have used the Hazard Index to evaluate the health risks of contaminated groundwater around the Kuwait’s Bay (Alsabti et al. 2022). They found that 13 of 19 samples had potential health risks on humans. Based on the calculations of hazard quotient (HQ) in the semi urban region of South India, severe F^−^ and NO_3_^−^ health risks have been identified (Dhakate et al. 2023).

In recent years in parallel with the increasing impacts of climate change and rapid expansion of water-intensive and polluting production processes such as battery production for electromobility, the population has become increasingly concerned about the quantitative and qualitative condition of groundwater resources in Hungary. Since the issue of water quantity and quality is currently of great interest to the public, therefore the monitoring of changes is of paramount importance. In addition, with the rise in environmental awareness, the protection of water resources has now become a priority for rural communities. Deeper integration between science and society could contribute to solve the nitrate pollution problems affecting rural regions of the world (Morris et al. 2003). Citizen Science approach may provide a very good example of this direction. Brockhage et al. (2022) were able to carry out thousands of nitrate measurements involving hundreds of residents and students in Germany, not only collecting a large amount of data, but also gaining the attention and active participation of the public (Brockhage et al. 2022).

Sanitation services are essential for ensuring public health and environmental protection, particularly in built-up areas, since untreated wastewater pose significant risk and can cause irreversible damage to aquatic systems (Barros 2023; Samudro et al. 2023). For wastewater management, on-site and off-site systems can be used in urban and rural areas, depending on the population size and financial conditions (Ghangrekar 2022). In the case of on-site systems, wastewater is stored within the boundaries of the building yard, typically in septic tanks or cesspits (Reay 2004; Richards et al. 2016). The adverse environmental impacts of these systems have been demonstrated by numerous studies (Mester et al. 2019; Withers et al. 2014). As a consequence, efforts to support the deployment of off-site systems have become a high priority in recent decades. In accordance with the European Union (EU) Council Directive concerning urban wastewater treatment (91/271/EEC), the establishment of the sewer systems of municipalities with pollutant loads above 2000 inhabitant equivalents (IE) is a compulsory task of the member states. Accordingly, a number of investments have been carried out in Hungary and are still projects in progress, raising the proportion of households connected to the sewer system to 88.1% by 2022 (Hungarian Central Statistical Office n.d.).

Urbanisation, particularly the expansion of sewerage networks, has a profound impact on groundwater quality. Since investments result in significant changes in the environment within the municipalities and can mitigate nitrate pollution by preventing the infiltration of untreated wastewater, it is of particular importance to monitor the ongoing changes. While several studies focus on the immediate impacts of urbanisation on water quality, and the majority of scientific works deal with analyses of current conditions, only few investigate the long-term dynamics of nitrate reduction following sewerage network construction. In addition, long-term and comprehensive monitoring is only feasible in very few cases.

This study aims to evaluate changes in nitrate concentrations by comparing pre- and post-sewerage water quality conditions, while also assessing the long-term effectiveness of nitrate attenuation following the sewerage installation. This approach enhances the understanding of the long-term impacts of sewerage network construction on groundwater nitrate levels. In addition, GIS techniques and statistical analyses will be used to provide a detailed overview of the spatial distribution of nitrate pollution. By this approach, it offers a more comprehensive understanding of how urbanisation-related infrastructure influences groundwater quality, with a particular focus on nitrate contamination. The results of the study can serve as useful and beneficial scientific information and can provide information for local citizens and authorities to improve groundwater management tools in the region.

## Materials and methods

### Geographical and geological description of the study area

**Fig. 1:**
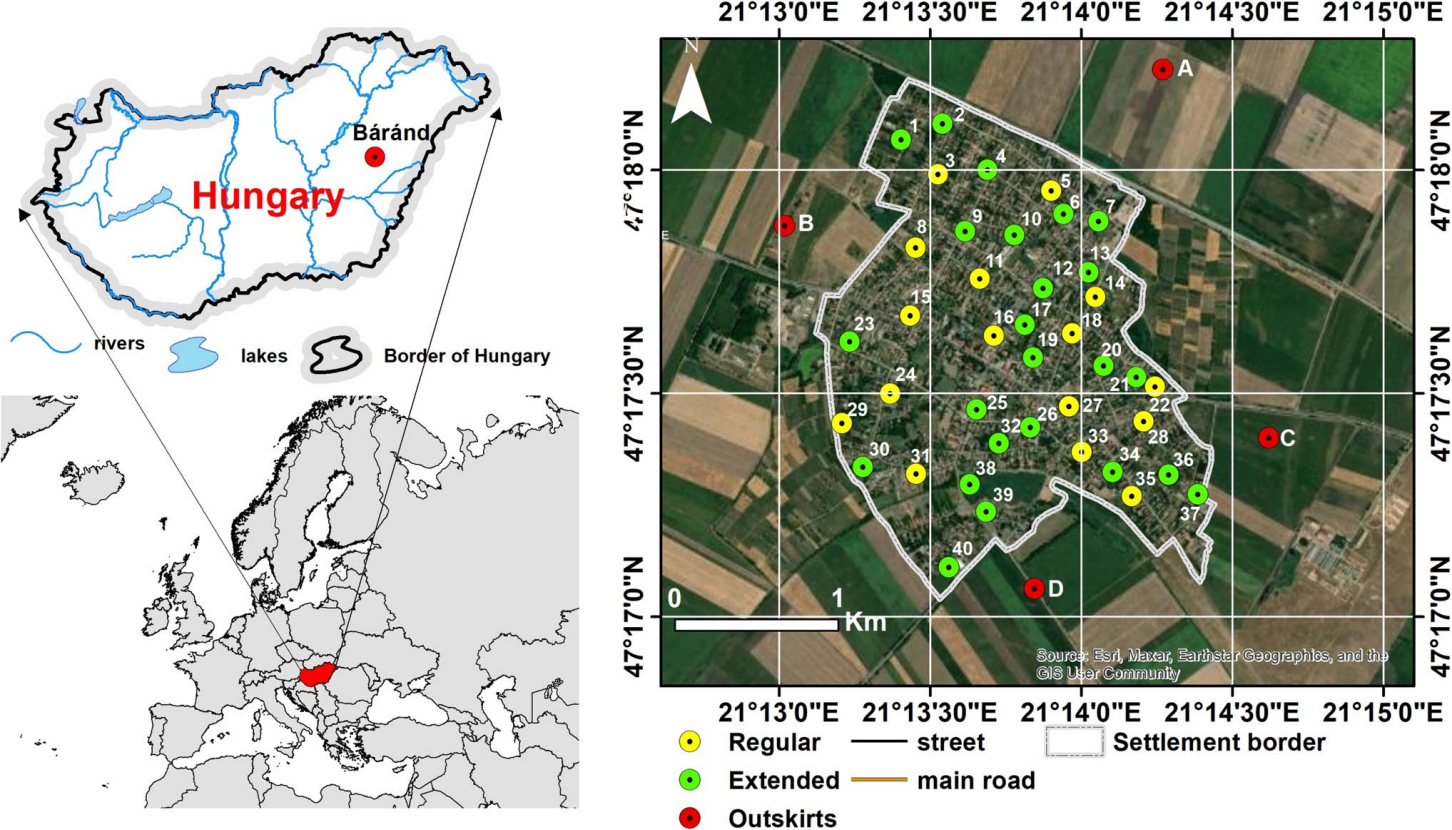
Location of the study area and the monitoring wells

**Fig. 2:**
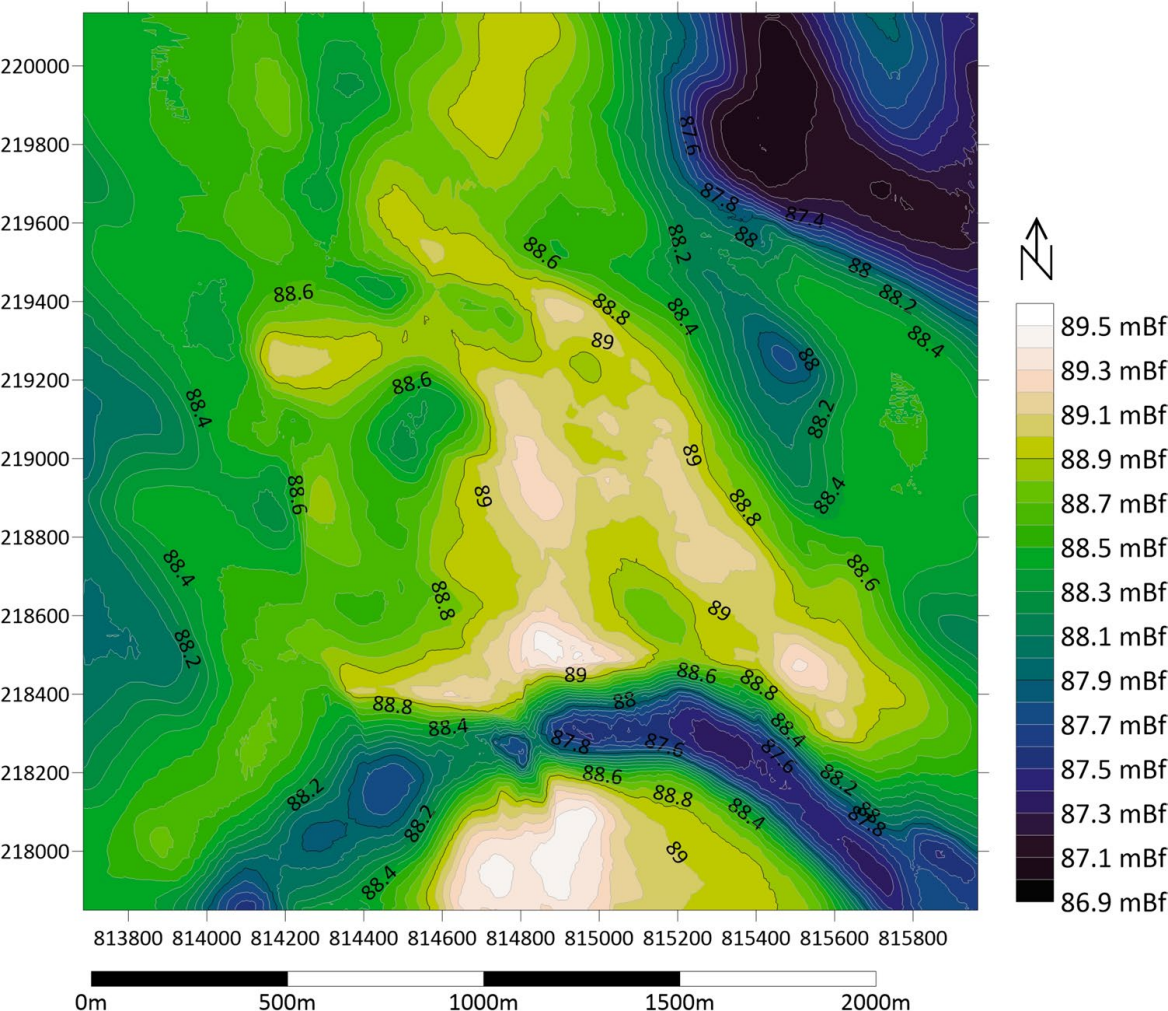
Digital elevation model (DEM) of the study area

The examined village of Báránd is situated in the eastern region of the Great Hungarian Plain, specifically in the Nagy-Sárrét area on the western side of the Sebes-Körös River’s alluvial deposit (Fig. 1). The population of the settlement was 2426 with a decreasing trend in 2023. The elevation of the Nagy-Sárrét area generally ranges from 85 to 89 m, and it is characterised as a flat plain with a relative relief of 0–3 m/km^2^. The digital elevation model created for the settlement and its surroundings is shown in Fig. 2. The direction of regional groundwater flow is oriented from south to north. The groundwater level is close to the surface, typically at a depth of 1–5 m, leading to the development of various soil types influenced by water (Michéli et al. 2006). Within the study region, prevalent soil types include Solonetz, Vertisol, Kastenozem and Chernozem, while in the settled area, anthropogenic effects have resulted in the formation of Anthrosols and Technosols.

The water sampling site is located within a settlement area, characterised by significant anthropogenic activities. According to the CORINE Land Cover (CLC) classification, the site falls under the “111” continuous urban fabric category. This indicates a densely built-up area with a predominance of impervious surfaces, such as residential buildings and infrastructure.

The housing stock of the settlement is 1135, of which only 74 lack basic utilities (HSCO, 2023). In 2022, the number of houses connected to the sewage network was 939 (HSCO, 2023). Over the past decade, the annual water production in the settlement has ranged from 90,000 to 120,000 m^3^, with a corresponding supply of 70,000 to 90,000 m^3^ to households. Our calculations suggest that approximately 40–60% of municipal wastewater stored in cesspits without proper insulation may have seeped into the soil, resulting in an estimated annual leakage of 30,000–55,000 m^3^ at the municipal level during the period prior sewerage. The sewer network of the settlement was constructed in 2014 in compliance with relevant legislation, and more than 80% of the dwellings have been connected. In 2022, the amount of drinking water supplied to households was 75,800 m^3^, while the volume of wastewater discharged to a treatment plant was 59,010 m^3^ (HSCO 2023).

**Fig. 3:**
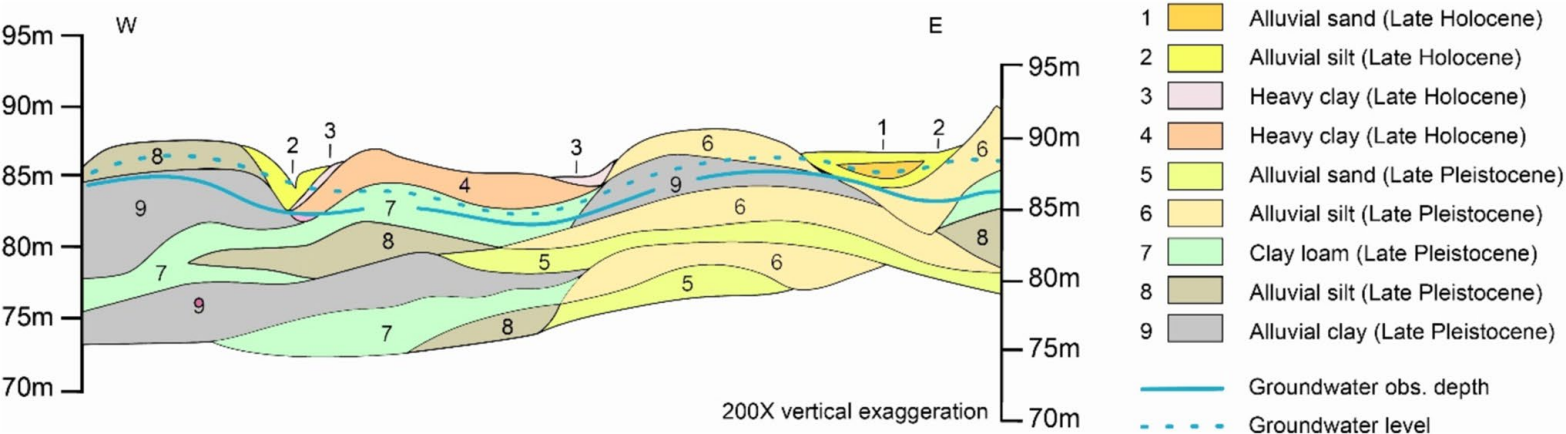
Geological cross section of the study area (modified based on Rónai 1980)

According to the shallow geological cross section of the settlement, Late Holocene and Lace Pleistocene sediments are dominant at the study area (Fig. 3). All deposits are of fluvial origin, the texture is predominantly clay and silt; however, alluvial sand layers can be observed at a depth of around 5 m. The high clay content plays an important role in terms of ammonium fixation and nitrate accumulation, which has been confirmed by our measurements (Mester et al. 2020a, b). High fixation capacity slows down the clean-up processes, as large amounts of organic and inorganic pollutants have accumulated in the soil over the last decades. At the same time, in terms of vertical spreading of contaminants, it is more favourable than sandy soils.

### Sampling and laboratory analysis

Regular sampling of selected groundwater wells in the study area was carried out between 2011 and 2023 (Fig. 1). Depths of the monitoring wells ranged from 6 to 10 m. From 2013, the number of wells was increased to 40 during the summer sampling period in order to determine the spatial extent of contamination with the highest possible accuracy (Fig. 1). During water sampling, the upper 1-m section of the water column was sampled and transported in airtight flasks to the Laboratory of Geosciences at the University of Debrecen, for further analysis. Electrical conductivity (EC) and pH of the water samples were determined using WTW 315i and CONSORT C3010 measuring instruments. Dissolved oxygen (DO) was measured by Aquaread AP5000.

In order to assess the impact of agricultural activities on groundwater quality in the vicinity of the settlement, we established four monitoring wells in the outskirts of the settlement (Fig. 1), from which samples were taken in 2013. However, we were unable to maintain the wells in the long term due to the agricultural activity.

The most characteristic pollutants of municipal wastewater were included in the analysis. The ammonium ion (NH_4_^+^), nitrite ion (NO_2_^−^), nitrate ion (NO_3_^−^), orthophosphate ion (PO_4_^3−^), chloride ion (Cl^−^) and chemical oxygen demand (COD) concentrations were determined according to the Hungarian Standards (HS ISO 7150–1:1992, HS 448–18:2009, HS 1484–13:2009, HS 12750–17:1974). The sodium ion (Na^+^) was determined by using a PERKIN ELMER 300 atomic absorption device. Three replicates of each sample were analysed to ensure reproducibility and minimise variability in the results. Certified standards were employed for quantification, and instruments were calibrated as per the required protocols to maintain accuracy. These steps were taken to ensure the reliability and validity of the analytical data obtained. The results were assessed on the basis of the relevant limits of the *Joint* Decree *No 6/2009*. (IV. 14.) *KvVM*-*EüM*-*FVM*.

### Applied GIS techniques and statistical analysis

IBM SPSS 26 software was utilised for the statistical processing of the data series and visualisation of the results. Beyond computing fundamental statistical metrics such as mean, lower and upper quartiles, mode, median and standard deviation, an assessment of the series’ normality was conducted. To enhance clarity, the results were depicted on various scatterplot and boxplot diagrams. Boxplot diagrams were chosen for their effectiveness in illustrating interquartile range, median and extremal values. Spearman’s rank correlation test was employed to assess the strength of the relationship between the variables. A hierarchical cluster analysis (HCA) was carried out to explore differences of spatial variation in nitrate concentrations. One-way analysis of variance (one-way ANOVA) assesses the variation (the range of scores) among different groups and contrasts it with the variability within each individual group. In order to test the temporal differences in NO_3_^−^ concentrations prior and after the sewerage network construction, the following null hypothesis is framed: H_0_: There is no significant difference in the pre-sewerage and post-sewerage periods on the NO_3_^−^ concentrations. A one-way ANOVA analysis was conducted on the data series over years (2011–2022) to accept or reject the null hypothesis, during which the Levene test was used to assess homogeneity of variance. Tukey and Games-Howell post hoc tests were also conducted. When performing the hierarchical cluster analysis (HCA), Euclidean distance and Ward’s method were applied as the similarity measurement and linkage, respectively.

GIS applications play a crucial role in assessing the spatial distribution of groundwater quality parameters by integrating spatial data with other geographical information (Nath et al. 2024). The visualisation of results and the spatial distribution of NO_3_^−^ were determined through inverse distance weighting (IDW) interpolation using ArcGIS 10.4.1 software. In environmental sciences, interpolation serves as a fundamental geostatistical analysis method, frequently employed to predict values in unobserved locations based on observed values in specific locations (Li and Heap 2014). Interpolation techniques are grounded in the principle that points in close proximity share greater correlations and similarities than those at a further distance. The inverse-distance weighting (IDW) method, a deterministic model for spatial interpolation, stands is widely used by geoscientists (Fischer et al. 2021; Makaya and Maphosa 2023; Naik et al. 2022). Its popularity is, in part, attributable to its widespread implementation in numerous GIS packages (Lu and Wong 2008). This implies that the extent of correlations and similarities between neighbouring points is directly proportional to the distance that separates them. This distance is conceptualised as a reverse function, depicting the relationship between every point and its neighbouring points (Setianto and Triandini 2015).

$${Z}_{0}=\frac{\sum_{i=1}^{N}{z}_{i}\times {d}_{i}^{-n}}{\sum_{i=1}^{N}{z}_{i}\times {d}_{i}^{-n}}$$

where *Z*_*0*_ is the estimation value of variable *z* in point *I*. The value of the sample at point *I*. is denoted as *z*_*i*_. *d*_*i*_ is the distance of sample point to estimated point; *N* is the coefficient that determines weigh based on a distance and *n* is the total number of predictions for each validation case.

### Nitrate pollution index

Concentrations of nitrate below the contamination limit of 50 mg/L in a particular sample do not signify that no anthropogenic impact can be detected in that same sample. Literature data show that values above 20 mg/L indicate the presence of human influences (Obeidat et al. 2012). The Nitrate Pollution Index (NPI) considers this value in the calculation, thus providing a more detailed overview of pollution levels; therefore, it has been a commonly used method for assessing nitrate pollution (Arslan and Çolak 2023; Panneerselvam et al. 2020; Venkata Ratnalu et al. 2023). However, identifying natural background levels for groundwater in urbanised areas is crucial but challenging due to the extensive human activities that alter the natural hydrochemical baseline. The variability in local geology, hydrology and historical land use also adds to the difficulty, requiring comprehensive long-term monitoring and advanced analytical techniques to accurately assess groundwater quality. Since the study area is under strong anthropogenic influence, it is challenging to determine the natural background level. Therefore, for calculating the NPI, we used the literature value of 20 mg/L.

Since the Nitrate Pollution Index (NPI) indicates whether human activity has contributed to nitrate pollution of groundwater, therefore it was also applied to provide more detailed information regarding NO_3_^−^ contamination in the study area using following formula (Obeidat et al. 2012):

$$NPI=\frac{{C}_{s}-HAV}{HAV}$$

where *C*_*s*_ is the nitrate concentration of each sample, and *HAV* (human affected value) is the threshold value of anthropogenic origin set at 20 mg/L according to Obeidat et al. (2012). NPI is classified into five groups as shown in Table 1.

**Table 1:** Values and categories of NPI and WHO limits on NO_3_^−^ (Almasri 2007; Obeidat et al. 2012; WHO 2004)

NO_3_^−^ (mg/L)	NPI value	NPI class	NPI interpretation	NO_3_^−^ (mg/L) WHO limit	WHO interpretation	NO_3_^−^ class
< 20	< 0	1	Clean (unpolluted)	< 50	Desirable limit	1
20–40	0–1	2	Light pollution	= 50	Maximum permissible limit (MPL)	2
40–60	1–2	3	Moderate pollution	> 50	Not permissible limit (NPL)	3
60–80	2–3	4	Significant pollution			
> 80	> 3	5	Very significant pollution			

### Health risk assessment

Health risk assessment is considered as one of the most effective approaches for determining the risk to human health from environmental pollutants (Wongsanit et al. 2015). The assessment of non-carcinogenic risks associated with nitrate-contaminated drinking water intake utilised the human health risk assessment methodology established by US EPA (U.S. Epa 1989). The evaluation was conducted across various age groups, including infants (0–2 years), children (2–6 years), teenagers (6–16 years) and adults (≥ 16 years). It is important to highlight that, as per US EPA guidelines, nitrate is classified as a non-carcinogenic risk factor for human health. In the exposure assessment, the calculation of chronic daily intake (CDI) (mg/kg/day) of nitrate from drinking water was performed using the methodology outlined in the guideline:

$$\mathrm{CDI}=\frac{\mathrm{C}\times \mathrm{IR}\times \mathrm{ED}\times \mathrm{EF}}{\mathrm{BW}\times \mathrm{AET}}$$

Table 2 presents the definitions and values of the parameters used in the formula.

**Table 2:** Parameters applied to calculate the chronic daily intake (CDI) and hazard quotient (HQ) of nitrate in the groundwater (Sailaukhanuly et al. 2024)

No	Parameter	Unit	Infant (0 ≤ 2 years	Child (2 ≤ 6 years)	Teenager (6 ≤ 16 years)	Adults (≥ 16 years)
1	C, concentration	mg/L	Measured in the study area			
2	IR, ingestion rate	L/day	0.62	0.78	2.0	2.5
3	ED, exposure duration	year	1	6	16	30
4	EF exposure frequency	day/year	350	350	350	350
5	BW, body weight	kg	11.4	18.6	56.8	80
6	AET, average exposure time	day	365	2190	5840	10950
7	RfD, reference dose	mg/kg day	1.6	1.6	1.6	1.6

A toxic effect is expected if the exposure dose of the pollutant exceeds the reference dose, usually expressed as a hazard quotient (HQ). RfD represents the reference dose for nitrate exposure, signifying a measure of chronic non-carcinogenic risks (1.6 mg/kg/day) (IRIS 2012). If the hazard quotient (HQ) exceeds 1, it signifies that the non-carcinogenic risk surpasses the acceptable level, suggesting a potential health risk.

$$\mathrm{HQ}=\frac{\mathrm{CDI}}{\mathrm{RfD}}$$

## Results and discussion

### Descriptive analysis of nitrate concentrations over 2011–2022

Data series for NO_3_^−^ concentrations between 2011 and 2022 were examined on an annual basis. In order to assess the temporal and spatial changes caused by the construction of the sewerage network in the municipality in 2014, the data series were divided into two periods: (1) pre-sewerage period (2011–2014) and (2) post-sewerage period (2015–2022). One of our key objectives when assessing the results was to identify what measurable changes have resulted from the investment.

Descriptive statistics on the annual evolution of nitrate concentrations are shown in Table 3 presenting the statistical values for the pre- and post-sewerage periods. According to the results, severe contamination of the shallow groundwater was found in the municipality. Data from years before sewerage show concentrations significantly higher than the WHO and Hungarian limit of 50 mg/L. The upper quartile value of NO_3_^−^ exceeded 500 mg/L in the years 2011 and 2014, while in 2012 the value was 497.8 mg/L. The lower quartile value exceeded 50 mg/L in all years (LQ range, 50.1–183.4 mg/L) prior to sewerage. However, the positive trend is clearly indicated by the decrease in the post-sewerage period. For the 7-year data series after the investment, the lower quartile values ranged between 23.7 and 43.1 mg/L and exceeded 50 mg/L only in 2022 (LQ = 76.1 mg/L). A significant decrease was observed in the upper quartiles, with values ranging from 153.0 to 377 mg/L between 2016 and 2022, representing a decrease of about 50% compared to the previous period.

**Table 3:** Descriptive statistics on the annual evolution of nitrate concentrations in mg/L

Nitrate	2011		2012	2013	2014	2015	2016	2017	2018	2019	2021	2022
Mean	459.2		332.3	187.8	378.2	231.1	155.5	142.6	109.7	170.7	164.5	296.1
Median	486.7		352.8	104.6	266.3	65.6	50.2	73.6	45.9	120.6	87.9	172.2
Std. Deviation	306.9		241.1	164.3	322.9	260.3	159.1	159.2	129.5	171.7	181.7	329.9
Variance	94193		58129	27020	104285	67774	25322	25375	16774	29509	33024	108835
Range	926.4		791.4	562.4	1057.1	669.1	439.1	612.1	531.3	637.8	812.5	1176.4
Minimum	52.28		23.3	2.36	29.1	2.62	20.1	4.46	6.95	7.61	16.5	9.64
Maximum	978.7		814.7	564.8	1086.2	671.7	459.2	616.6	538.3	645.5	829.1	1186.0
Percentiles	25	183.4	113.5	50.1	96.7	33.2	34.9	37.6	23.7	43.1	33.2	76.1
	50	486.7	352.8	104.6	266.3	65.6	50.1	73.1	45.9	120.6	87.9	172.2
	75	623.2	497.9	341.7	535.9	509.1	302.8	221.1	153.0	244.8	184.9	377.3

**Fig. 4:**
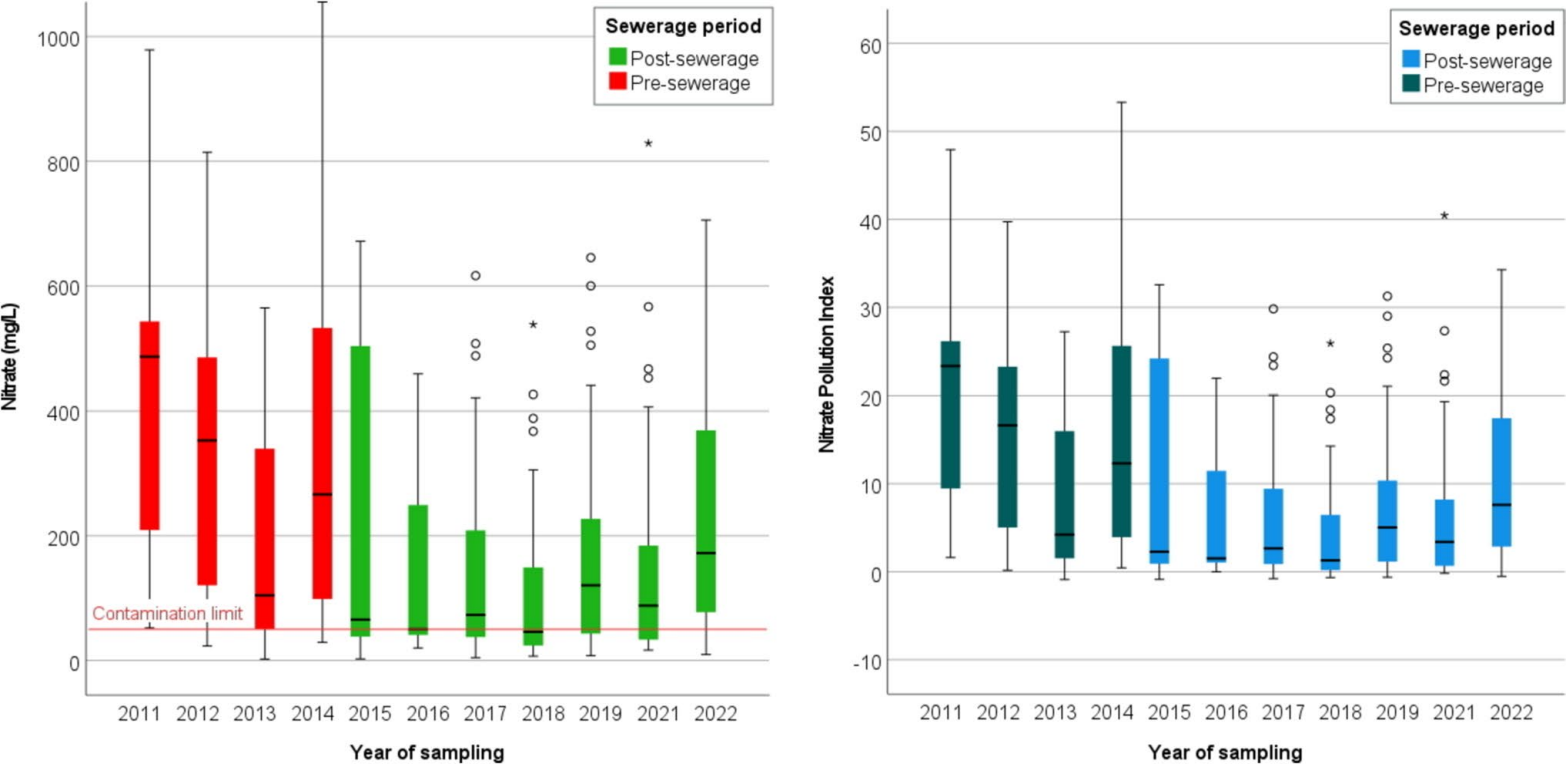
Nitrate concentrations and Nitrate Pollution Index values between 2011 and 2022

The positive trends are also illustrated by the boxplot charts (Fig. 4). The year 2015 (UQ = 509.1 mg/L) is still very similar to previous years, which can be explained by the fact that only 1 year has passed since the investment. Elevated nitrate values in 2022 may be related to the fact that 2022 was the driest year over the last decades, which significantly affected groundwater levels and evaporation processes in wells; however, further investigations are required to confirm this relationship, and the potential impact of climate change on groundwater dynamics should also be assessed.

Descriptive statistical tests carried out for the pre- and post-sewerage conditions provide a simplified and more comprehensive picture of the differences between the two periods. The mean NO_3_^−^ concentration decreased from 289.7 to 175.6 mg/L, the upper quartile from 475.5 to 233.5 mg/L. The increase in the number of wells with concentrations below the limit value is indicated by the decrease in the lower quartile from 70.8 to 38.5 mg/L.

The extremely different contamination conditions of the monitoring wells are revealed by the fact that the difference between the highest (1086.2 mg/L) and lowest (2.36 mg/L) value for the pre-sewage period was more than 1000 mg/L. This difference has slightly increased in the period following sewerage, indicating that clean-up processes are relatively slow and that other local effects can still have a strong influence on the contamination status of the monitoring wells.

High NO_3_^−^ concentrations in the shallow groundwater resources of the settlement are in line with the results of other studies carried out in semi-urban and rural areas (Jawadi et al. 2022; Rahman et al. 2021). Adhikary et al. (2012) measured nitrate levels ranging from 57 to 1923 mg/L in the peri-urban areas of New Delhi, India, and identified wastewater as a major cause of the alarming levels of pollution. Su et al. (2020) found elevated levels of NO_3_^−^ in the shallow groundwater beneath residential areas of Muling-Xingkai Plain, China, with a clear connection to anthropogenic activities dating back to the 1950s. The increased NO_3_^−^ concentrations (8.5–251 mg/L) were attributed to domestic sewage discharge.

### Origin of nitrate in shallow groundwater

Chloride ions (Cl^−^) play a crucial role in assessing wastewater-induced groundwater contamination, as their presence is independent of microbiological and physical processes. Cl^−^ derived from chemical fertilisers typically lead to a notable elevation in NO_3_^−^ concentrations; conversely, markedly high Cl^−^ levels accompanied by relatively low NO_3_^−^ levels often signify the influence of domestic sewage, industrial wastewater and livestock effluent (Wei et al. 2017). Hence, analysing the NO_3_^−^/Cl^−^ ratios can provide more insight into the dynamics and sources of nitrogen (Torres-Martínez et al. 2021). Thus, the dual-logarithm diagram has been applied in numerous studies to identify the origin of groundwater nitrate (NO_3_^−^) concentrations. He et al. (2022) and colleagues investigated the natural and anthropogenic factors, and among other measurement based on the NO_3_^−^/Cl^−^ ratios found, that manure and sewage were the main contributors to high NO_3_^−^ levels. Widory et al. (2005) and colleagues concluded in their studies in selected sample sites in France that high Cl^−^ values associated with lower NO_3_^−^ values indicate sewage effluent.

**Fig. 5:**
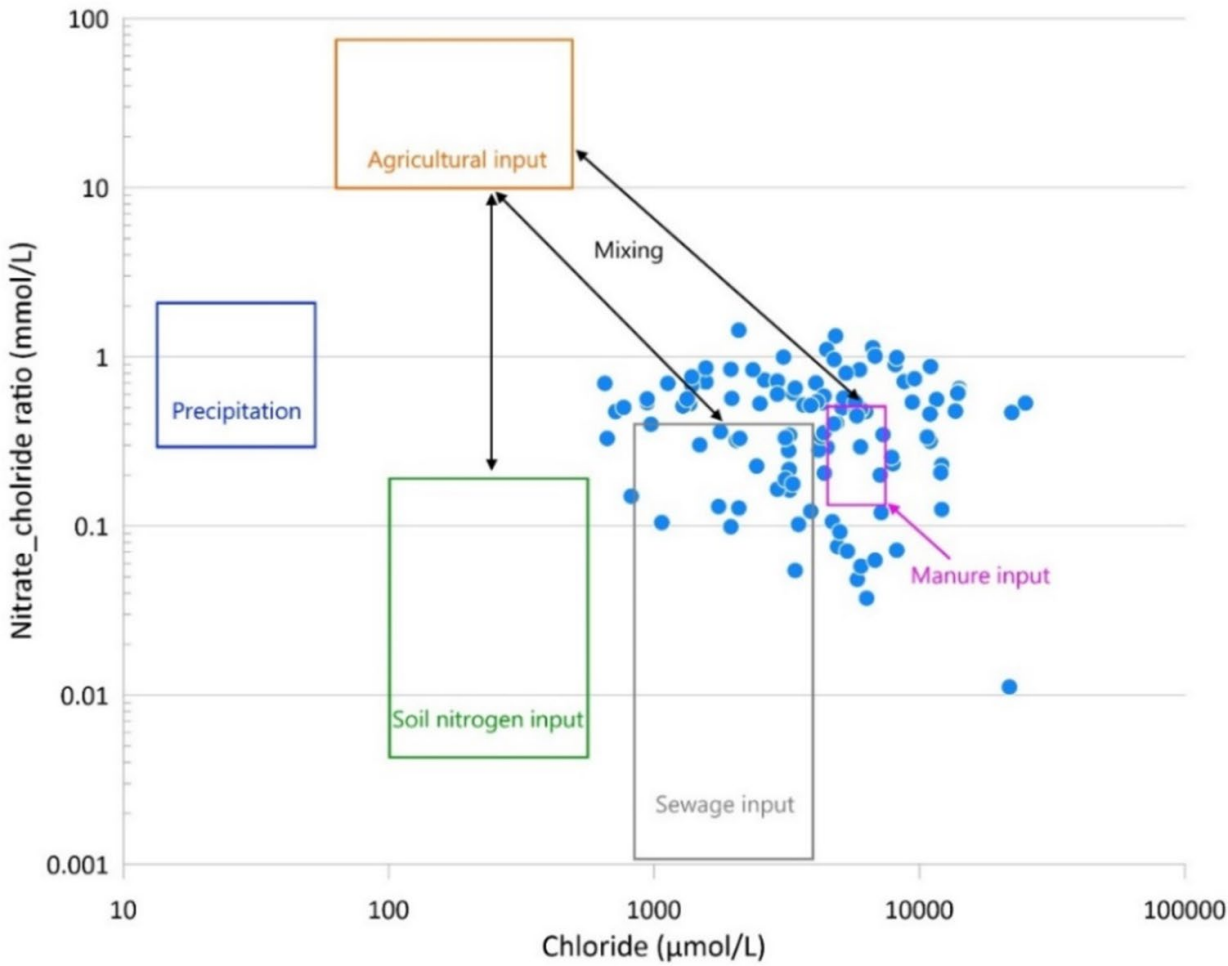
Identification of NO_3_^−^ source: dual-logarithm diagram of NO_3_^−^/Cl^−^ molar ratios vs. Cl^−^ (sampling year 2019)

Variations in NO_3_^−^/Cl^−^ molar ratios versus Cl^−^ molar concentrations are presented in Fig. 5. The result suggest that the contamination of shallow groundwater primarily resulted from anthropogenic sources, including septic tank effluent from households and the extensive use of manure as an organic fertiliser. Slight mixing with agricultural activity is also observed, but the impact of sewage and manure is predominant.

**Fig. 6:**
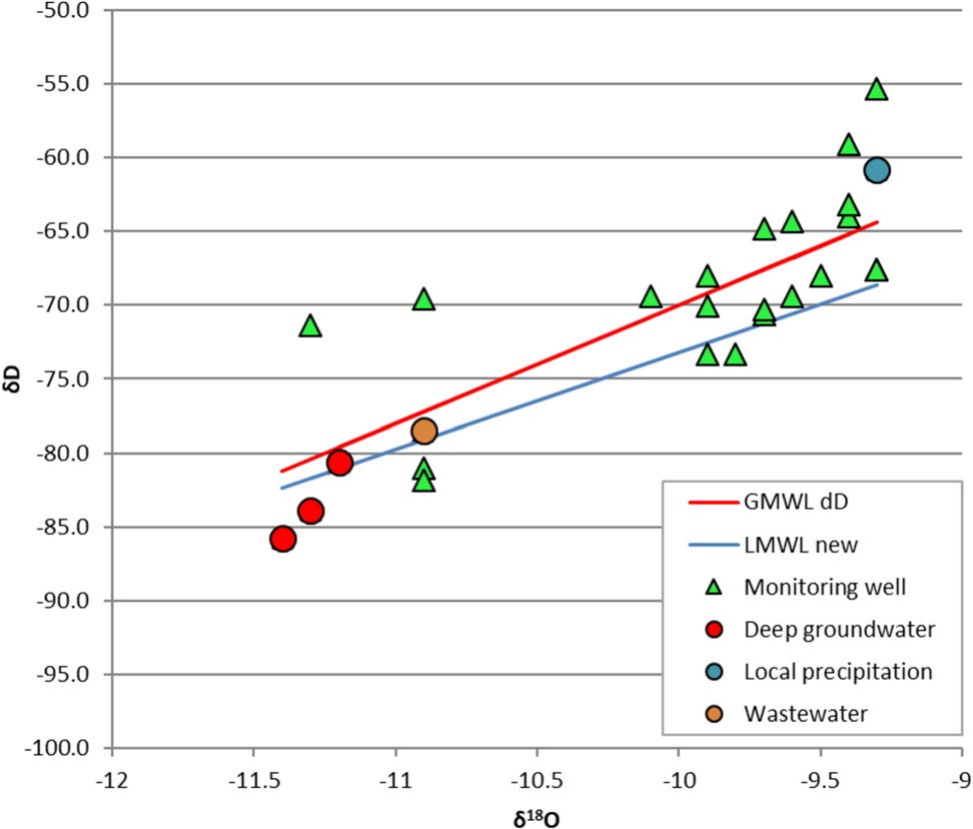
δD and δ^18^O values of the monitoring wells in selected monitoring wells, 2013

To verify the presence of wastewater discharges in the monitoring wells of the municipality, the isotopic ratio shifts (δ) for ^18^O and D(^2^H) were determined for the year 2013. The δD values are plotted against δ^18^O values, and the global (GMWL) and local precipitation line (LMWL) are denoted (Fig. 6).

As the isotopic ratios of wastewater and precipitation from groundwater differ significantly, it is possible to detect wastewater mixing with groundwater. In the majority of the wells, the δ^18^O and δD values were significantly lower than the values of the local precipitation (δ^18^O =  − 9.3; δD =  − 60.8). The results obtained confirm our hypothesis that the groundwater in the monitoring wells is affected by municipal wastewater. The local precipitation line (LMWL) additionally enables the assessment of whether evaporation or recharge predominantly influences a specific sample. This suggests that evaporation can be observed in the majority of samples (Fig. 6).

**Fig. 7:**
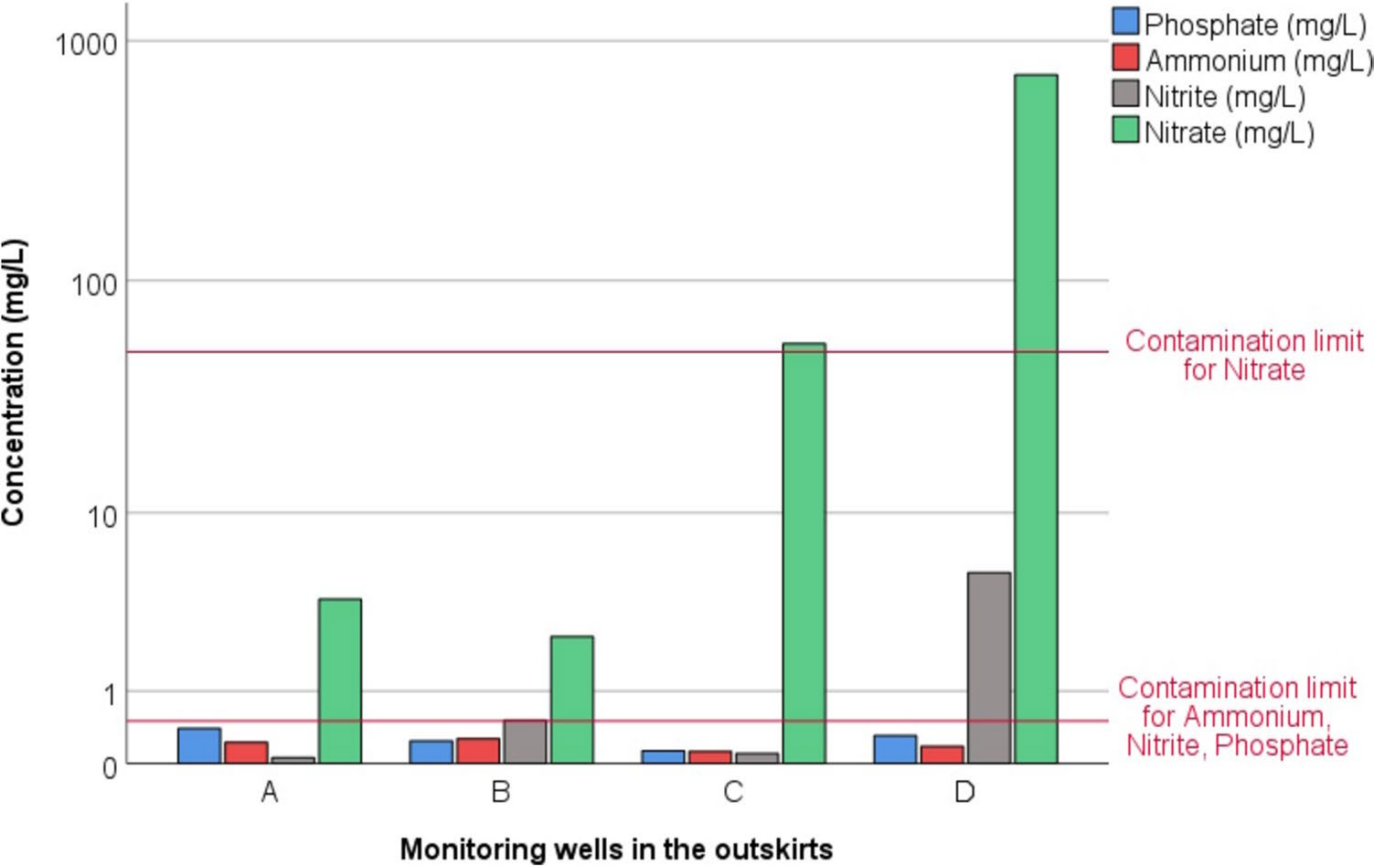
The concentration of inorganic nitrogen forms and phosphate in the monitoring wells outside the settlement in 2013

In order to assess the agricultural impact on NO_3_^−^, we established monitoring wells in agricultural areas outside the settlement and collected water samples (Fig. 1). Figure 7 shows the concentrations of phosphate, ammonium, nitrite and nitrate. Wells A and B, located in the northern and western directions, show low NO_3_^−^ values (2.5 and 3.7 mg/L); in contrast, nitrate concentrations are significantly elevated in the southern (well C) and south-eastern (well D) wells, especially in well D, where nitrate reaches nearly 729 mg/L. This increasing pattern in nitrate contamination aligns with the regional groundwater flow direction, which moves from north to south, indicating a potential transport of nitrate contaminants along the flow gradient. Although the ammonium and phosphate concentrations do not exceed the contamination limits, the elevated values clearly reflect the influence of agricultural activities in the area.

### Nitrification processes in the study area

The dissolved oxygen (DO) concentration determines the type of microbial processes present in the groundwater and also influences the completeness of the reaction steps (Nikolenko et al. 2018). Particularly in the case of anaerobic conditions, microbial nitrification processes are unlikely, whereas denitrification processes prevail under such conditions (Goldberg et al. 2008). It has also been reported that pH values below 5.5 enhance nitrous oxide (N_2_O) accumulation, likely because N_2_O reductase is mostly inhibited under acidic conditions enabling N_2_O accumulation in the subsurface environment; thus, the denitrification process does not continue to the final step (Deurer et al. 2008). It has also been found that denitrification can result in the highest N_2_O at moderate DO concentrations (below 3.15–4 mg/L), since most denitrifiers are facultative anaerobes (Deurer et al. 2008).

**Fig. 8:**
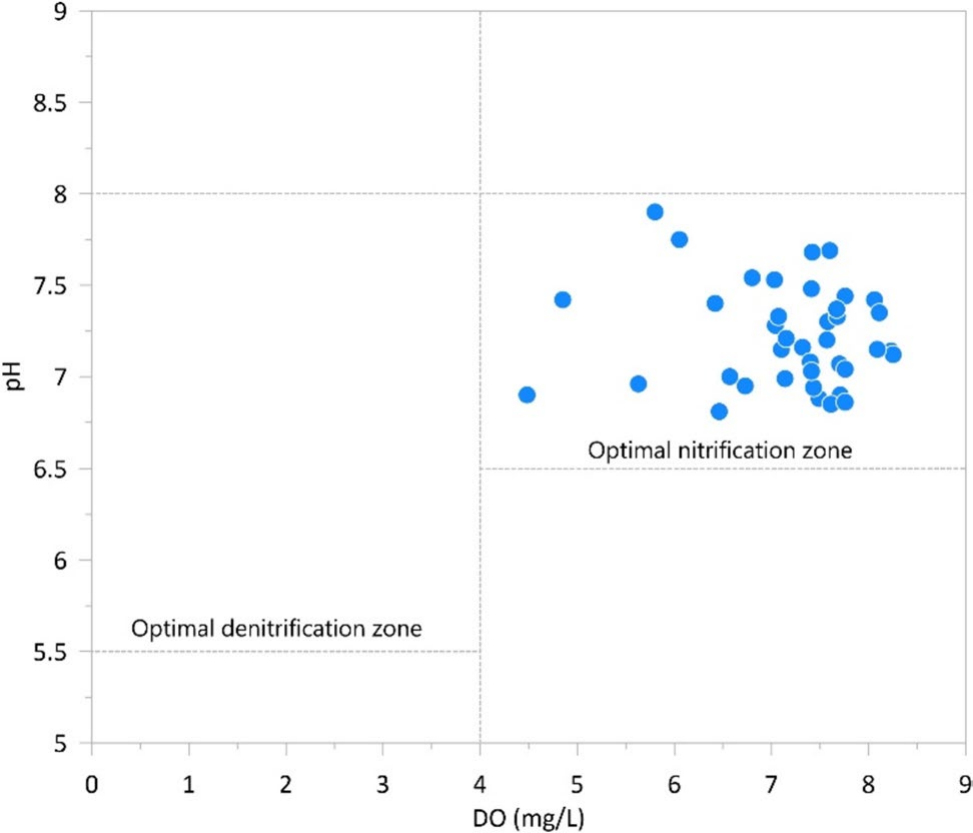
Scatterplot contrasting pH with DO; the dashed lines determine the optimal zones of nitrification and denitrification processes

Since pH and DO have a combined effect on nitrification processes, the optimal zone for nitrification and denitrification processes can be defined (Rathnayake et al. 2015; Torres-Martínez et al. 2021). This is illustrated in Fig. 7, where the pH versus DO values are plotted for the year 2019. The figure clearly shows that all samples fell into the ideal zone for nitrification, which resulted in higher NO_3_^−^ concentration. DO values of the samples varied from 4.48 to 8.25 mg/L, while pH ranged from 6.81 to 7.9 (Fig. 8). It can be concluded based on the results that the nitrification process contributes significantly to nitrate concentrations.

### Correlation of NO_3_^−^ with other hydrochemical parameters

For the study period 2011–2022, continuous data are available for eight parameters, whereas for the other parameters, unfortunately, data are not continuously collected throughout the years. Table 4 shows the results of the Spearman correlation analysis for the regularly measured parameters. It was revealed that the strongest significant (*p* < 0.01) positive correlation with NO_3_^−^ has the electrical conductivity (EC) with a value of 0.710 in the pre-sewerage and with 0.669 in the post-sewerage period. In addition, a strong correlation was found for NO_3_^−^/Na^+^ (*r* = 0483) in the pre-sewerage period, which decreased to 0.248 in the post-sewerage period. In the post-sewerage period in the case of NO_3_^−^/NH_4_^+^, significant positive correlation was found, and a negative correlation in the case of NO_3_^−^/pH (*r* =  − 0.339).

**Table 4:** Spearman correlation matrix of the hydrochemical parameters for the pre- and post-sewerage periods

Pre-sewerage period								
Parameter	pH	EC	NH_4_^+^	NO_2_^−^	NO_3_^−^	PO_4_^3−^	COD	Na^+^
pH	1							
EC	− 0.268*	1						
NH_4_^+^	− 0.10	0.409**	1					
NO_2_^−^	0.21	0.483**	0.363**	1				
NO_3_^−^	− 0.265*	0.710**	0.209	0.256	1			
PO_4_^3−^	0.102	0.108	− 0.108	− 0.143	− 0.022	1		
COD	0.108	0.281*	0.281	0.271*	− 0.037	0.418**	1	
Na^+^	− 0.123	0.448**	0.145	0.220	0.483**	0.079	0.001	1
Post-sewerage period								
Parameter	pH	EC	NH_4_^+^	NO_2_^−^	NO_3_^−^	PO_4_^3−^	COD	Na^+^
pH	1							
EC	− 0.231**	1						
NH_4_^+^	− 0.046	0.469**	1					
NO_2_^−^	− 0.069	0.353**	0.286**	1				
NO_3_^−^	− 0.339**	0.669**	0.372**	0.282**	1			
PO_4_^3−^	0.381**	− 0.134	0.050	− 0.030	− 0.072	1		
COD	0.196**	0.035	0.286**	0.139*	− 0.038	0.321**	1	
Na^+^	− 0.008	0.470**	0.122	− 0.250**	0.248**	− 0.068	0.243**	1

*Correlation significant at the level 0.05 (two-tailed)

**Correlation significant at the level 0.01 (two-tailed)

**Fig. 9:**
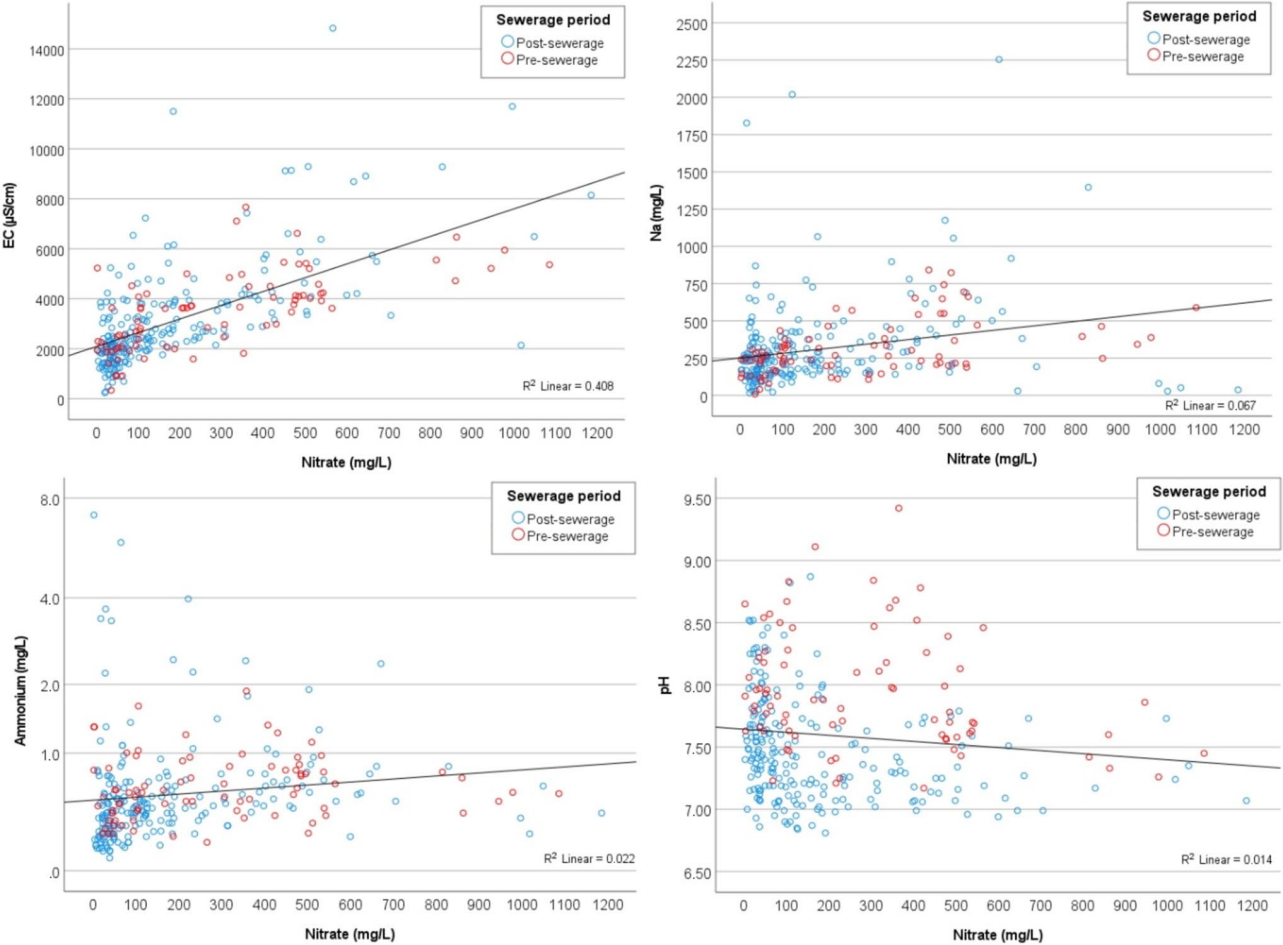
Scatterplot diagrams of NO_3_^−^/EC, NO_3_^−^/Na^+^, NO_3_^−^/NH_4_^+^ and NO_3_^−^/pH values for the pre- and post-sewerage periods

In order to provide a more detailed view of the relationship between the parameters, NO_3_^−^/EC, NO_3_^−^/Na^+^, NO_3_^−^/NH_4_^+^ and NO_3_^−^/pH values were plotted against each other divided for pre- and post-sewerage period (Fig. 9). A significant decrease in pH can be seen in the period after sewerage, the mean value decreased from 7.94 to 7.46. In addition, EC values also showed a decreasing trend, with the upper quartile falling from 4497 to 3858 µS/cm and the lower quartile from 2155 to 1867 µS/cm. NH_4_^+^ values are also found to be in a significantly lower range, the upper quartile decreased from 0.82 to 0.65 mg/L, while the lower quartile decreased from 0.42 to 0.30 mg/L. Although high Na^+^ concentrations (> 1000 mg/L) were also measured after the canalisation, the lower quartile was reduced from 200.6 to 151.3 mg/L. The high values are the result of several factors, in addition to the geological and soil conditions in the area, Na^+^ was also influenced by increased evaporation due to the drought in 2022.

### Spatial distribution and changes of NO_3_^−^ over time

**Fig. 10:**
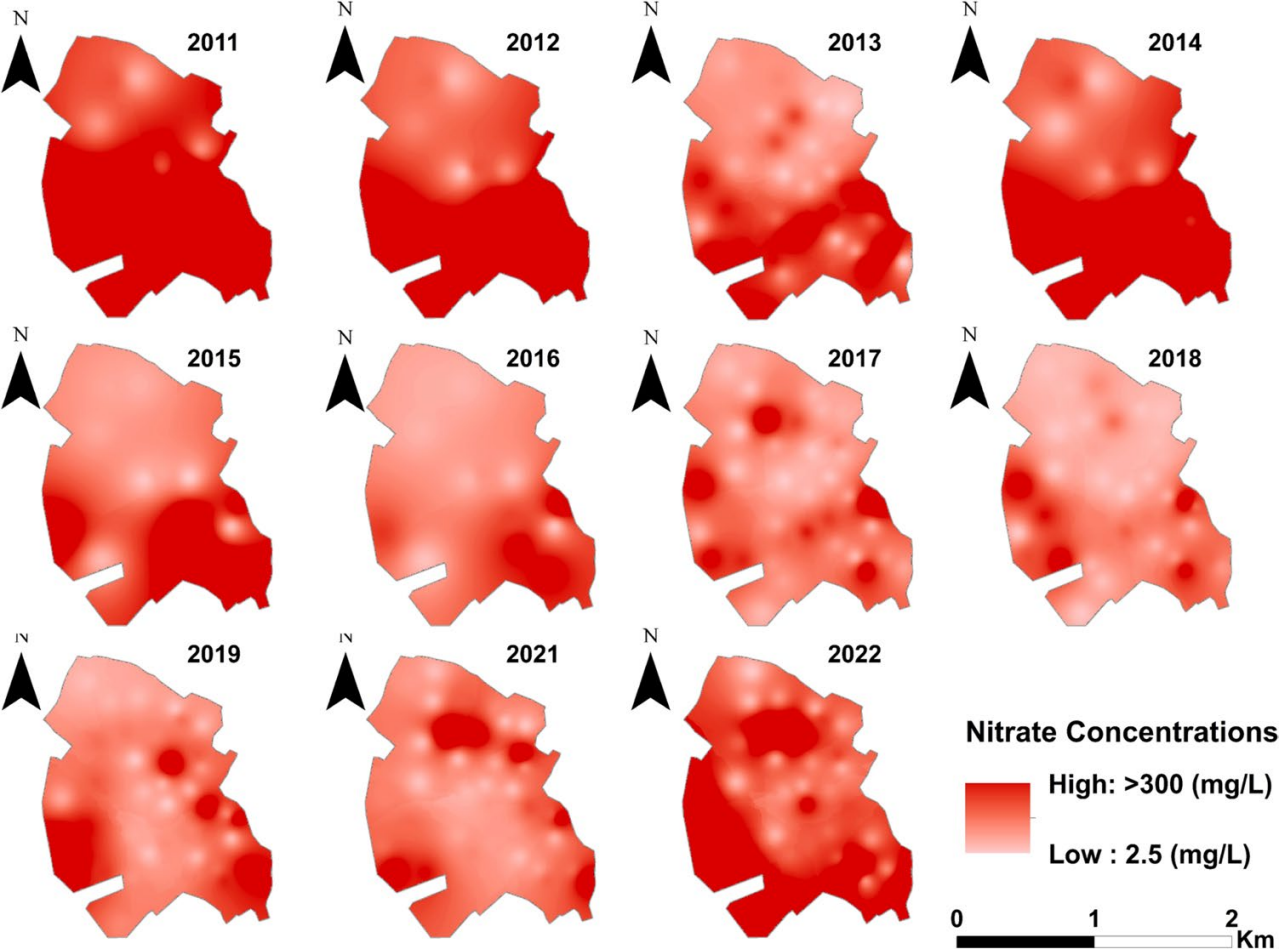
Annual NO_3_^−^ distribution maps for the pre-sewerage (2011–2014) and post-sewerage (2015–2022) period

To reveal the spatial evolution of NO_3_^−^ in the study area, interpolated spatial distribution maps were created for all years investigated between 2011 and 2022 (Fig. 10). The interpolated maps also show temporal changes of spatial patterns in the pre-sewerage (2011–2014) and post-sewerage (2015–2022) period. The time-series maps show a clear distinction between the considerably higher pollution levels in the period before sewerage. For the period 2011–2014, NO_3_^−^ concentrations several times above the relevant limit value of 50 mg/L are observed in most parts of the municipality, accompanied by a N-S increase in pollution.

In 2015, the year after sewerage, the N-S pattern is very similar to previous years; however, the northern parts of the settlement can be considered less polluted. In the period 2016–2021, the proportion of areas with lower levels of pollution increased markedly, with a parallel decrease in the N-S disparity, and concentrations above 300 mg/L were only observed in the SE and NW parts of the settlement (Fig. 10).

Elevated nitrate concentrations in 2022 may be attributed to an extremely severe drought; however, further investigations are needed to confirm this. This finding is supported by the long-term (2009–2018) groundwater nitrate studies by Johson and colleagues in Scotland, which concluded that nitrate concentrations are highly sensitive to meteorological variations (Johnson et al. 2023). Lindsey and colleagues also found a strong relationship between nitrate and periods with drier or wetter than average based on thirty years of regional groundwater quality trend analysis in the USA (Lindsey et al. 2023). Both regional and local scale studies have revealed that recharge events commonly result in dilution of groundwater NO_3_^−^ levels (Opsahl et al. 2017).

The results indicate the ongoing clean-up processes, but also highlight that groundwater pollution caused by anthropogenic nitrogen is potentially a long-term problem, as nitrogen storage in the vadose zone can last for decades (Mester et al. 2020a, b; Meter et al. 2016). This conclusion is consistent with the findings of other studies (Burow et al. 2010; Wassenaar et al. 2006). With the elimination of sources of pollution, a decrease in NO_3_^−^ starts, which can be seen in our study. Kyte and colleagues observed a significantly decreasing temporal trend in selected wells located in parcels where manure application had ceased more than a decade earlier (Kyte et al. 2023).

**Fig. 11:**
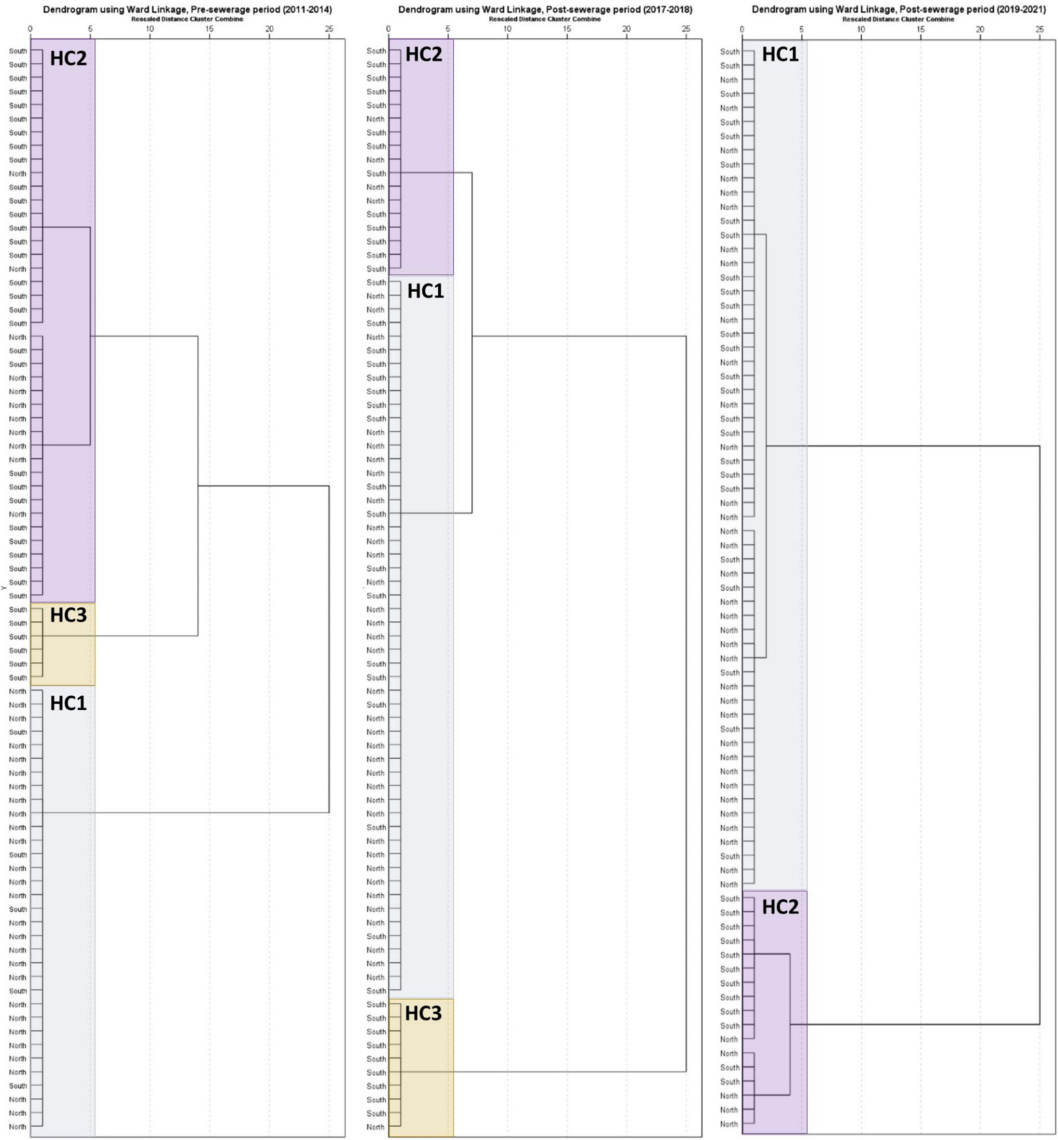
Ward’s dendogram of the hierarchical cluster analyses, based on the location of the monitoring well in the pre- and post-sewerage period

Due to the apparent N-S differences in nitrate levels, a hierarchical cluster analysis was performed, dividing the northern and southern areas (20–20 monitoring wells in each category). Due to the large number of samples, the analysis was performed for 3 time intervals: (1) the entire period before the sewerage, which includes 80 measurements; (2) the post-sewerage period, with data from the years 2017 and 2018; and (3) the post-sewerage period, with data from the years 2019 and 2021 (Fig. 11).

The results of the pre-sewerage period clearly show a cluster of 33 wells (HC1) with predominantly northern wells (76%), a cluster (HC2) of 41 wells with a 73% share of southern wells and a cluster (HC3) of exclusively southern wells. In the second period considered, the size of the clusters containing predominantly southern wells decreased, indicating a reduction in the differences. This process continues in period 3, where only a single and relatively small cluster of predominantly southern well was found. In the dominant cluster, northern and southern wells were mixed (58% vs 42%).

### Nitrate pollution index

The boxplot diagrams of calculated NPI values are presented in Fig. 4. The annual percentage distribution of samples within the 5 NPI categories is shown in Table 5. Out of a total data set of 297 samples for the period 2011–2022, only 22 samples were classified as “clean”, of which 82% were collected in the post-sewerage period. 15.8% of the total sample was classified as “light polluted” with an increasing frequency in the post-sanitation period as well. Fifty-eight percent of the samples were classified in the category “very significant pollution”; however, the percentage shows a downward trend after the sewerage (Table 5).

**Table 5:** Crosstabulation of the NPI values for the years

			Year											Total
			2011	2012	2013	2014	2015	2016	2017	2018	2019	2021	2022	
NPI	Clean (unpolluted)	Count	0	0	4	0	2	0	5	7	2	1	1	22
		% within NPI	0.0%	0.0%	18.2%	0.0%	9.1%	0.0%	22.7%	31.8%	9.1%	4.5%	4.5%	100.0%
		% within year	0.0%	0.0%	10.0%	0.0%	15.4%	0.0%	12.5%	17.5%	5.0%	2.7%	2.9%	7.4%
		% of total	0.0%	0.0%	1.3%	0.0%	0.7%	0.0%	1.7%	2.4%	0.7%	0.3%	0.3%	7.4%
	Light pollution	Count	0	2	2	2	2	3	9	9	6	10	2	47
		% within NPI	0.0%	4.3%	4.3%	4.3%	4.3%	6.4%	19.1%	19.1%	12.8%	21.3%	4.3%	100.0%
		% within year	0.0%	15.4%	5.0%	15.4%	15.4%	23.1%	22.5%	22.5%	15.0%	27.0%	5.9%	15.8%
		% of total	0.0%	0.7%	0.7%	0.7%	0.7%	1.0%	3.0%	3.0%	2.0%	3.4%	0.7%	15.8%
	Moderate pollution	Count	1	0	6	0	1	4	4	7	6	1	2	32
		% within NPI	3.1%	0.0%	18.8%	0.0%	3.1%	12.5%	12.5%	21.9%	18.8%	3.1%	6.3%	100.0%
		% within year	7.1%	0.0%	15.0%	0.0%	7.7%	30.8%	10.0%	17.5%	15.0%	2.7%	5.9%	10.8%
		% of total	0.3%	0.0%	2.0%	0.0%	0.3%	1.3%	1.3%	2.4%	2.0%	0.3%	0.7%	10.8%
	Significant pollution	Count	0	0	4	0	3	0	5	1	2	3	4	22
		% within NPI	0.0%	0.0%	18.2%	0.0%	13.6%	0.0%	22.7%	4.5%	9.1%	13.6%	18.2%	100.0%
		% within year	0.0%	0.0%	10.0%	0.0%	23.1%	0.0%	12.5%	2.5%	5.0%	8.1%	11.8%	7.4%
		% of total	0.0%	0.0%	1.3%	0.0%	1.0%	0.0%	1.7%	0.3%	0.7%	1.0%	1.3%	7.4%
	Very significant pollution	Count	13	11	24	11	5	6	17	16	24	22	25	174
		% within NPI	7.5%	6.3%	13.8%	6.3%	2.9%	3.4%	9.8%	9.2%	13.8%	12.6%	14.4%	100.0%
		% within year	92.9%	84.6%	60.0%	84.6%	38.5%	46.2%	42.5%	40.0%	60.0%	59.5%	73.5%	58.6%
		% of total	4.4%	3.7%	8.1%	3.7%	1.7%	2.0%	5.7%	5.4%	8.1%	7.4%	8.4%	58.6%
Total		Count	14	13	40	13	13	13	40	40	40	37	34	297
		% within NPI	4.7%	4.4%	13.5%	4.4%	4.4%	4.4%	13.5%	13.5%	13.5%	12.5%	11.4%	100.0%
		% within year	100.0%	100.0%	100.0%	100.0%	100.0%	100.0%	100.0%	100.0%	100.0%	100.0%	100.0%	100.0%
		% of total	4.7%	4.4%	13.5%	4.4%	4.4%	4.4%	13.5%	13.5%	13.5%	12.5%	11.4%	100.0%

**Fig. 12:**
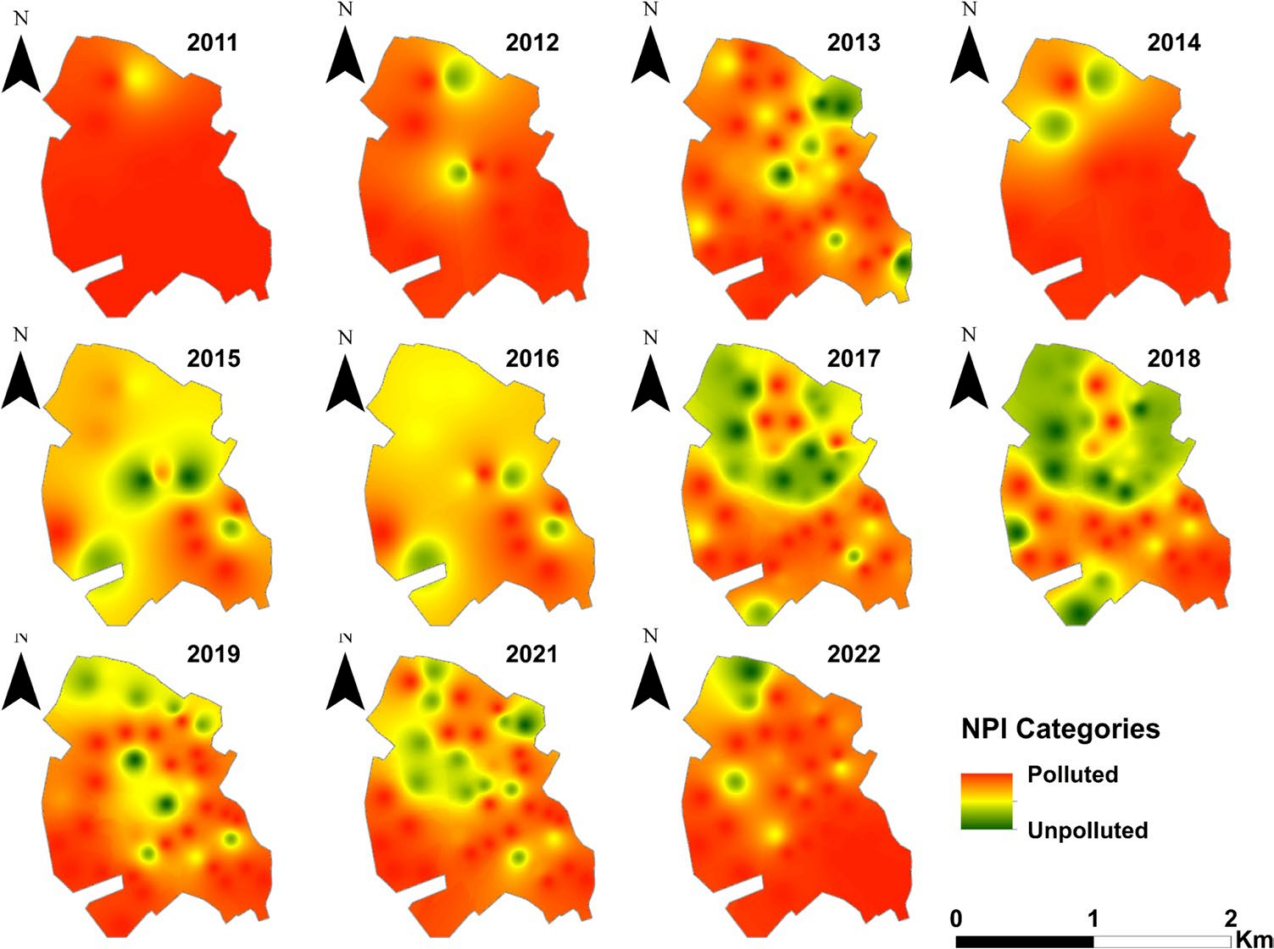
Annual NPI distribution maps for the pre-sewerage (2011–2014) and post-sewerage (2015–2022) period

Based on the NPI categories, time-series spatial distribution maps were produced, in order to identify the spatial patterns of the pollution (Fig. 12). The results also show that there are marked differences in the N-S direction and that the proportion of areas with lower pollution levels increases significantly after sewerage.

### Temporal trend analysis of nitrate concentrations

**Fig. 13:**
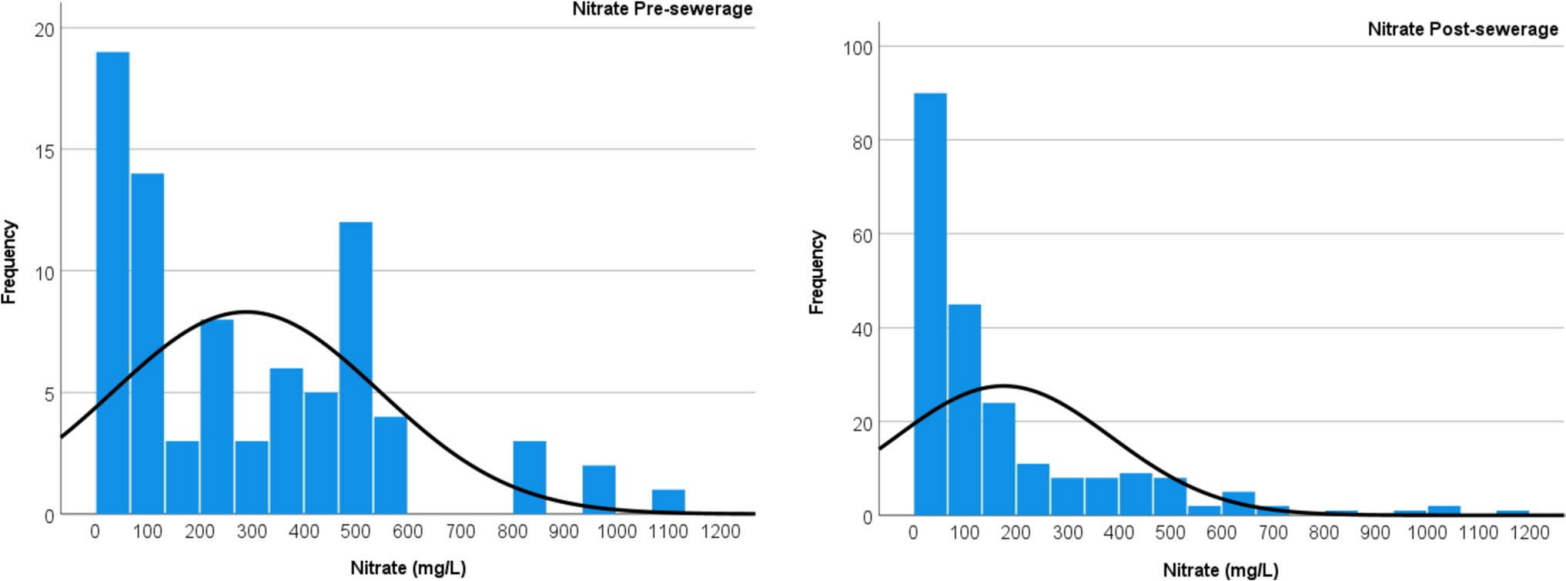
Frequency of NO_3_^−^ concentration in the pre- and post-sewerage period

The frequencies of nitrate concentrations for the pre- and post-sewerage periods are shown in Fig. 13. The most significant change was a significant increase in the frequency of relatively low concentrations below 100 mg/L, with a parallel marked decrease in the frequency of extremely high concentrations (> 500 mg/L).

**Fig. 14:**
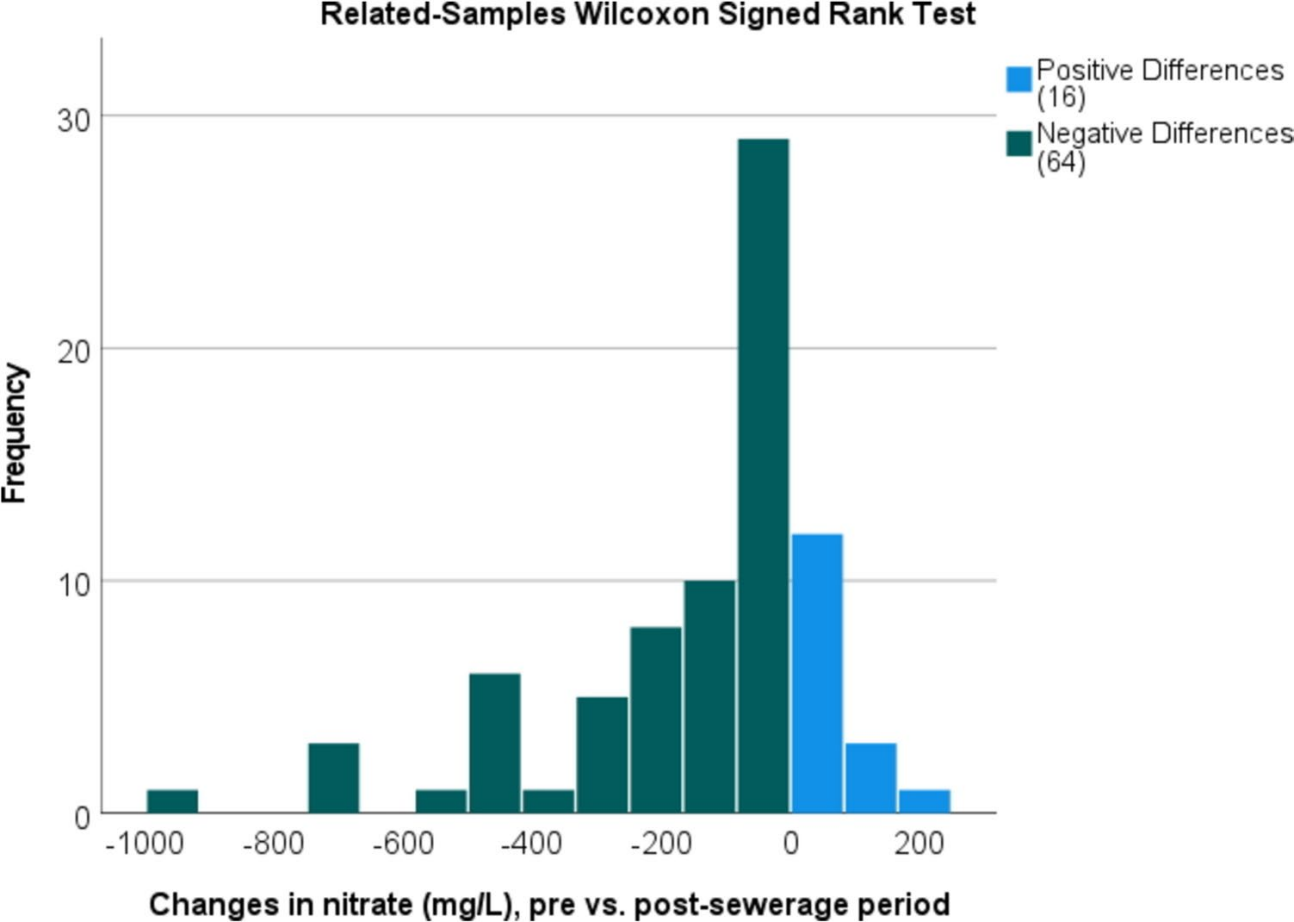
Frequency of positive and negative differences in NO_3_^−^ based on Wilcoxon signed rank test

Several studies have used the Wilcoxon signed rank test to determine the statistical difference between paired samples (Elçi and Polat 2011; Rupert 2008). In the case of our study area, the Wilcoxon signed rank test analysis was carried out by combining data from the years before and after sewerage for each individual well. According to the test, statistically significant differences (*p* value = 0.001) in NO_3_^−^ levels have been determined at 95% confidence level (*p* < 0.05). The frequency of positive and negative changes is illustrated in Fig. 14. The positive changes are illustrated by the fact that in 80% of all cases examined, a negative change was observed, i.e. a decrease in concentration compared to the reference year in the pre-sewerage period. In the vast majority of cases, a reduction of 0–50 mg/L was detected, with a decrease in frequency with increasing concentration value differences.

**Fig. 15:**
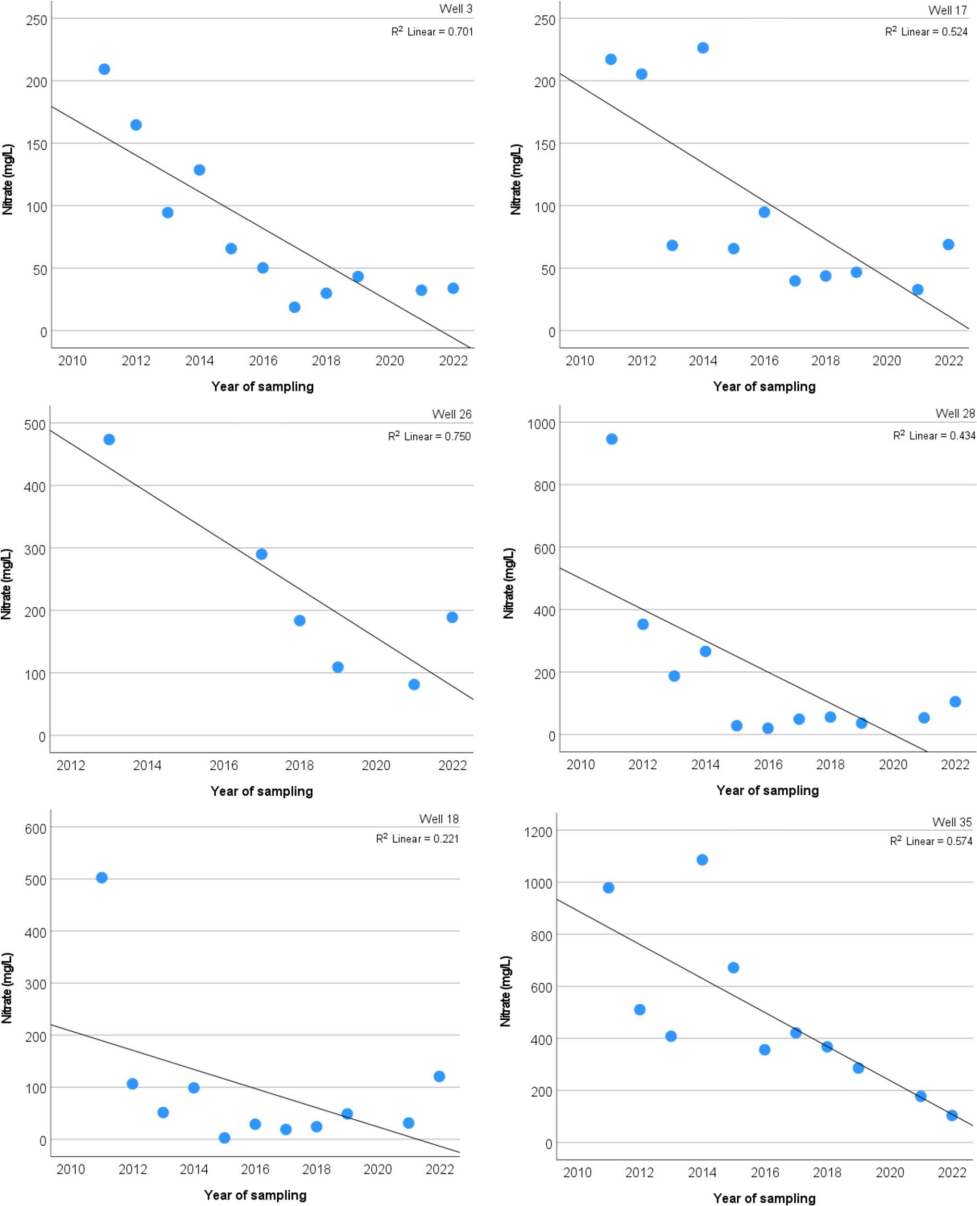
NO_3_^−^ trends over the period between 2011 and 2022 of selected monitoring wells

In order to show the significant decreasing trends more detailed, scatterplot diagrams were generated for 6 selected monitoring wells showing NO_3_^−^ concentrations as a function of the years of investigation (Fig. 15). In the case of the monitoring well 3, the values of 100–200 mg/L before sewerage were consistently dropped below 50 mg/L. Similar values and trends are observable in wells 17 and 28 respectively. A significant decrease from extremely high concentrations above 1000 mg/L to below 200 mg/L occurred in well 35.

One-way analysis of variance (ANOVA) using least significance difference (LSD) method was applied to test the significant differences in mean values of NO_3_^−^ concentration for the period 2011–2022. LSD is utilised to identify significant differences between group means as part of analysis of variance. There is always a difference between the mean values of a given parameter at different times, but whether the magnitude is statistically significant is uncertain. To perform the analysis, the results of the test of homogenity of variances are required, to decide whether the differences between the mean concentrations at the different sampling times are significant. As the significance value performed in SPSS software is lower than the 0.05 significance level, the null hypothesis assuming no difference in mean concentrations between sampling times is rejected. The result of the ANOVA shows a significant level at 0.001; hence, the NO_3_^−^ mean values at different sampling times are significantly different.

Numerous studies apply one-way ANOVA tests to detect differences between groups. (Chitsazan et al. 2017) and colleagues examined NO_3_^−^ concentrations at different sites using ANOVA and found significant differences between areas. The results of the ANOVA performed by Nemčić-Jurec and Jazbec (2017) showed statistically significant differences between groups of wells located at different distances from the point source of pollution (Nemčić-Jurec and Jazbec 2017).

The results from the long-term monitoring of NO_3_^−^ contamination in shallow groundwater of the municipality after the construction of the sewerage network provide crucial insights into the effects of sanitation investments on groundwater quality. Over the period of 12 years (2011–2022), this study has revealed that the construction of the sewer network significantly reduced nitrate contamination, though the cleanup process is slow and ongoing, as indicated by the gradual but steady decrease in NO_3_^−^ levels across the study area.

The substantial decrease in NO_3_^−^ concentrations post-sewerage underscores the effectiveness of sanitation interventions in reducing anthropogenic contamination. These results align with previous studies that report long-term improvements in groundwater quality following the installation of proper sanitation infrastructure (Hashmi et al. 2023).

However, despite the positive overall trends, some monitoring wells continue to exhibit high nitrate concentrations, with values exceeding 500 mg/L even in the post-sewerage period. This suggests that the decontamination process is not uniform across the study area, due to variations in previous contamination loads, and ongoing pollution sources, such as septic tank effluent and agricultural runoff (Widory et al. 2005). These findings are supported by the dual-logarithmic diagram of NO_3_^−^/Cl^−^ ratios, which implicates a combination of wastewater and manure as primary contamination sources. In addition, the retention of NO_3_^−^ in soils with high clay content plays a critical role in nitrate persistence (Adimalla and Qian 2023). Additionally, the role of climate variability, such as the observed spike in nitrate levels during the drought year of 2022, requires further research to assess how extreme weather events may affect groundwater quality over time (Lindsey et al. 2023). Continuous monitoring, coupled with targeted pollution reduction strategies, will be essential to achieve sustainable groundwater management and further decrease in NO_3_^−^ pollution.

### Health risk assessment based on hazard quotient

**Fig. 16:**
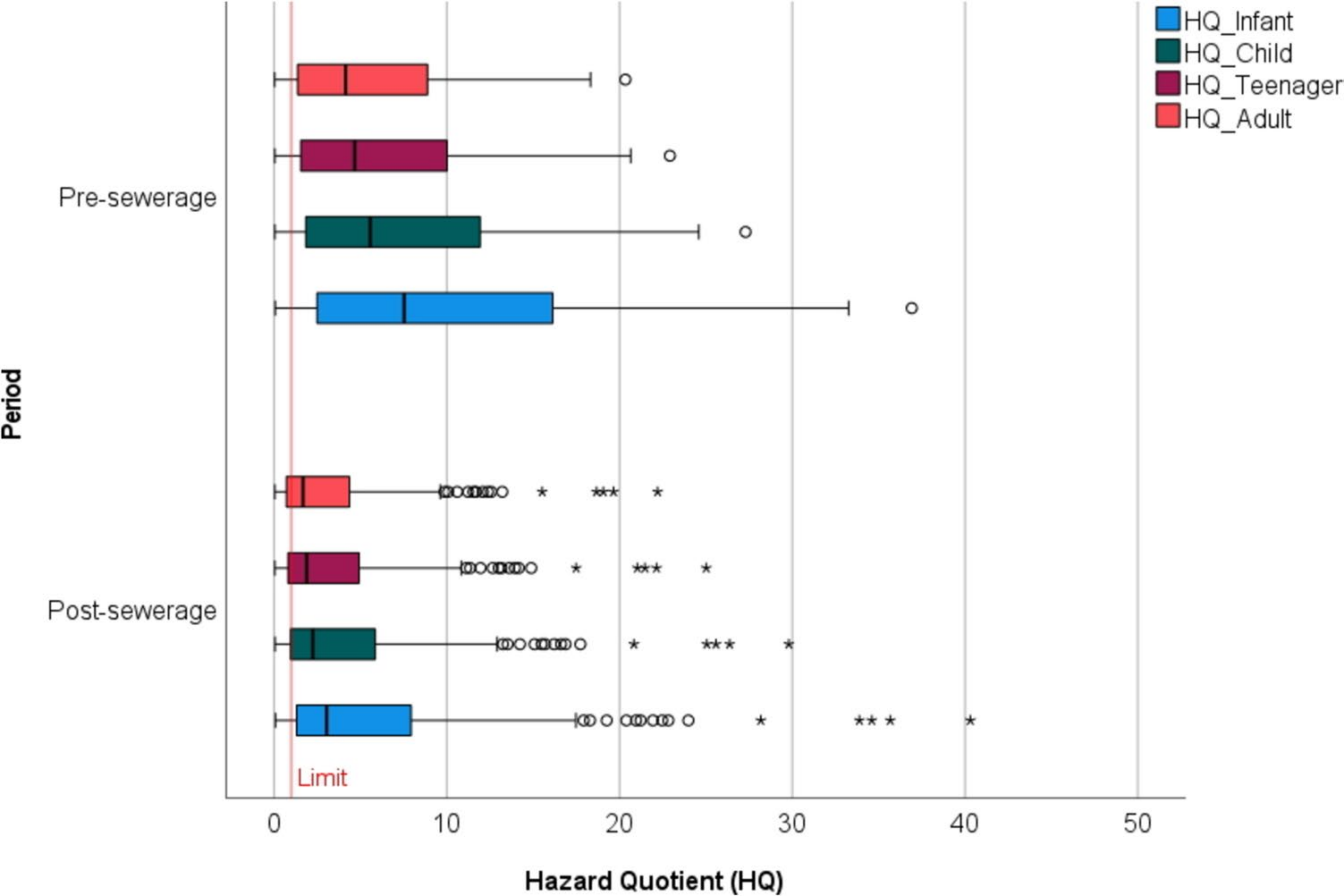
Results of human health risk assessment based on hazard quotient (US EPA)

The risk to human health was calculated based on the US EPA model and previously described parameters (Table 2). Since values higher than 1 indicate a health risk, the results indicate very high levels of potential risk. In the period before sewerage, significant health risk was identified for nitrate in the majority of the monitoring wells (Fig. 16). Upper quartile values exceeded the limit of non-carcinogenic risk by 10 times in the category teenager, 12 times in the category child and 17 times in the category infant. In the period after sewerage, a marked decrease occurred, the lower quartile values in the categories adult, teenager and child also decreased below the relevant limit (Fig. 16). Although the shallow groundwater is no longer used by the local population for water consumption, it should be noted that the water is consumed occasionally, especially by the older generation, and is also used for livestock watering, which can also pose a risk in the case of extremely high values. In accordance with our result, it has been demonstrated by numerous studies conducted in shallow aquifers that the consumption of shallow groundwater poses considerable health risks (Anim-Gyampo et al. 2019; Idriss et al. 2021; Wang and Li 2022; Wu and Sun 2016).

## Conclusions

The presence of sanitation systems is of paramount importance for the protection of the urban environment and human health. In the present study, a long-term monitoring (2011–2022) of shallow groundwater NO_3_^−^ contamination in municipal environment was carried over a period of almost a decade following the construction of the sewerage network in the light of the pre-sewerage situation.

Based on the results, significant pollution of the shallow groundwater in the municipality was identified. During the pre-sewer period, NO_3_^−^ concentrations exceeded the 50 mg/L limit in the majority of monitoring wells by a considerable margin. Using interpolated NO_3_^−^ pollution maps, differences in the spatial evolution of the contamination were also detected, characterised by marked N-S patterns, with a significant increase in NO_3_^−^ concentrations towards the south.

Variations in NO_3_^−^/Cl^−^ molar ratios suggest that the contamination of shallow groundwater primarily resulted from anthropogenic sources, including septic tank effluent from households and the extensive use of manure as an organic fertiliser. In order to verify the presence of wastewater discharges in the monitoring wells of the municipality, the isotopic ratio shifts (δ) for ^18^O and D(^2^H) were determined, confirming municipal wastewater effluent.

Data series of 7 years (2015–2022) after the investment indicate marked positive changes by the appearance of decreasing trends in NO_3_^−^ values. By comparing the pre- and post-sewerage conditions, considerable reduction of the NO_3_^−^ values was measured, with an increasing number of monitoring wells with concentrations below the limit.

According to Wilcoxon signed rank the test, statistically significant differences in NO_3_^−^ levels have been determined (*p* < 0.05). The positive trends are illustrated by the fact that in 80% of all cases examined, a decrease in concentration was detected compared to the reference year in the pre-sewerage period. In the vast majority of cases, a reduction of 0–50 mg/L was observed. Based on the variance analysis in the mean values of NO_3_^−^ concentration, significant differences were identified. By applying hierarchical cluster analysis (HCA), it has been shown that the significant N-S concentration difference is becoming less pronounced in the area.

Nitrate Pollution Index (NPI) was used to detect the presence of human influences. Out of a total data set of 297 samples for the period 2011–2022, only 22 samples were classified as “clean” (< 20 mg/L NO_3_^−^), of which 82% were collected in the post-sewerage period. Human health risk assessment, carried out according to the US EPA model, showed significant health risk for NO_3_^−^ in the majority of the monitoring wells with a decreasing trend during the post-sewerage period.

Our results highlight the importance of sanitation investments and the fact that the decontamination processes are particularly slow. Detailed, long-term monitoring is therefore essential to ensure accurate follow-up of the ongoing changes.

## Data Availability

The datasets generated during and/or analysed during the current study are available from the corresponding author on reasonable request.
